# Transcriptome sequencing and functional analysis reveal *RrCBF* genes as key regulators of cold adaptation in *Rosa rugosa*

**DOI:** 10.3389/fpls.2025.1732552

**Published:** 2025-12-10

**Authors:** Dongliang Guo, Mengqi Zhao, Yang Yang, Yushan Li, Leilei Zhu, Feifei Li, Haixia Jiang, Lin Xu, Liqiong Xie

**Affiliations:** 1 Xinjiang Key Laboratory of Biological Resources and Genetic Engineering, College of Life Science and Technology, Xinjiang University, Urumqi, China; 2 Crop Research Institute of Xinjiang Uygur Autonomous Region Academy of Agricultural Sciences, National Central Asian Characteristic Crop Germplasm Resources Medium-term Gene Bank, Urumqi, China; 3 Key Laboratory of Plant Stress Biology in Arid Land, College of Life Science, Xinjiang Normal University, Urumqi, China

**Keywords:** *Rosa rugosa*, cold stress, transcriptome sequencing, differentially expressed genes (DEGs), RrCBF transcription factors, heterologous overexpression

## Abstract

*Rosa rugosa* is an economically significant ornamental species with limited understanding of its molecular cold adaptation mechanisms. This study utilized transcriptome sequencing to elucidate the temporal dynamics and organ-specific regulatory mechanisms underlying cold stress (4°C) responses in the leaves and one-year-old stem of *R*. *rugosa*. Differential gene expression analysis revealed distinct organ-specific and time-dependent transcriptional reprogramming. A core set of 1,479 and 1,872 genes were consistently differentially expressed from early to late stages (4–24 h) in leaves and stems, respectively. Intersection analysis identified 1,550 conserved early cold-responsive genes shared between two *R*. *rugosa* cultivars. These genes were significantly enriched in the MAPK signaling pathway, plant hormone signal transduction, cytoskeleton-related processes, and metabolic reprogramming. Weighted gene co-expression network analysis (WGCNA) pinpointed *RrCBFs* as central hubs. Genome-wide characterization identifies five *RrCBF* genes in *R*. *rugosa* as cold-inducible central regulators, universally upregulated under cold stress despite divergent cis-elements. Heterologous overexpression of *RrCBF1*/*RrCBF5* in *Arabidopsis* enhanced freezing tolerance through reduced oxidative damage, improved osmoprotection, and stabilized photosystem function. Critically, transgenic lines exhibited pleiotropic developmental alterations: dwarfism, delayed flowering, and suppressed vegetative-reproductive transition, indicating trade-offs between growth and stress adaptation. Our results delineate a CBF-centric regulatory module coordinating antioxidant defense, photosynthetic protection, and developmental plasticity in *R*. *rugosa* cold adaptation, providing targets for cold-tolerance breeding.

## 1 Introduction

*Rosa rugosa* belongs to the genus *Rosa* L. in the family Rosaceae. The genus *Rosa* L., with roughly 200 species, is widely distributed across temperate and subtropical regions of the northern hemisphere. *R. rugosa* is widely cultivated across North America, Europe, and Eastern Asia, a popularity attributable to its attractive flowers (Chen et al., 2021). Its derivatives hold crucial positions in the cosmetics, food, and pharmaceutical industries, highlighting its multifaceted industrial value (Wang et al., 2023; Zhang et al., 2024a). The production and distribution of *Rosa* L. are severely hindered by environmental stresses, particularly low-temperature stress (Zhuang et al., 2021). Winter cold frequently causes a sharp increase in freezing damage rates for field-grown cut roses (*Rosa hybrida* L.), manifested as delayed flowering, diminished flower quality, and yield fluctuations (Schrock and Hanan, 1981). Furthermore, low temperatures inhibit root development and water uptake efficiency. They also impair photosynthetic capacity by reducing chlorophyll fluorescence parameters, ultimately resulting in growth retardation and diminished ornamental value (Chen et al., 2019). Additionally, the potential of *R. rugosa* to provide ecological services such as soil consolidation and urban greening in cold regions is constrained by its inability to adapt to these conditions. Therefore, understanding the molecular basis of cold tolerance in *R. rugosa* and developing hardy varieties are crucial for both expanding cultivation areas and securing supply chains, while also enhancing ecological resilience and ensuring sustainable economic benefits under climate change.

Low-temperature stress significantly inhibits plant growth by disrupting key cellular processes, including reduced cell membrane fluidity, dissociation of protein complexes, over-accumulation of reactive oxygen species (ROS), and decreased activity of various enzymes and photosynthetic rate (Allen and Ort, 2001; Yue et al., 2024; Kang et al., 2025). To cope with this challenge, plants have evolved multidimensional regulatory networks. These networks integrate pathways including light signaling, the circadian clock, plant hormone signaling, and pathogen defense, and accumulate protective compounds such as soluble sugars, proline, cold-regulated (COR) proteins, and detoxification enzymes to confer cold tolerance (Kidokoro et al., 2022; Kim et al., 2024; Kang et al., 2025). Within these networks, key transcription factors play crucial regulatory roles in the cold response. These include members of the AP2/ERF, MYB, WRKY, and NAC families (Zou et al., 2010; Diao et al., 2020; Lv et al., 2020; Xu et al., 2022; Wu et al., 2024). Among these cold-responsive regulatory networks, the CBF-COR module constitutes the core pathway for plant adaptation to cold stress (Qian et al., 2024; Roeder et al., 2025). In this pathway, low temperature signals rapidly induce the expression of *CBF/DREB1* (members of the AP2/ERF family). CBF proteins then specifically bind to the CRT/DRE elements in the promoters of *COR* genes, activating their expression to mediate plant adaptation to low temperatures (Shi et al., 2018; Kidokoro et al., 2022). Furthermore, the CBF pathway extensively crosstalks with light signaling and plant hormone signaling pathways (Magome et al., 2004; Shi et al., 2012; Jiang et al., 2017). These findings reveal that plant cold adaptation not only relies on the core CBF regulatory network but also achieves a dynamic growth-defense tradeoff through the integration of cross-pathway signals.

Recent studies reveal that rose cold resistance is governed by a multi-layered transcriptional regulatory network, with its core mechanism involves the coordinated response of cold signal perception, metabolic adaptation, and developmental plasticity. In the cold-responsive signaling pathway, the AP2/ERF superfamily plays a pivotal role, such as members of the CBF family (*RcCBF6* in *Rosa chinensis*), whose heterologous overexpression significantly enhances cold tolerance in *Arabidopsis* (Li et al., 2020). The functional conservation of the AP2/ERF family across species is evidenced by *Medicago truncatula* MtDREB1C, which activates DRE element-dependent cold resistance genes in *Rosa chinensis* (Chen et al., 2010). Subsequent research demonstrates that *RmERF54*, an ERF subfamily member in *Rosa multiflora*, directly enhances cold-hardiness phenotypes by reinforcing the DREB/COR signaling pathway; conversely, loss of *RmERF54* function leads to a drastic decline in freezing tolerance (Chen et al., 2024). Beyond the core cold-response pathways, the regulation of metabolic homeostasis also plays a pivotal role. For instance, the bHLH member RmICE1 from *Rosa multiflora* enhances cold tolerance in transgenic tobacco by promoting proline accumulation, reducing ROS through elevated antioxidant enzyme activity, and up-regulating stress-responsive genes (Luo et al., 2020). Concurrently, the Cys2/His2-type (C2H2) zinc finger protein RmZAT10 from *Rosa multiflora* contributes to maintaining intracellular homeostasis, which is achieved by specifically regulating proline biosynthesis and modulating ROS homeostasis, thereby playing a role in cold resistance (Luo et al., 2022). Furthermore, the cold stress response is deeply integrated with plant hormone signaling networks. For instance, in *Rosa persica*, specific bHLH transcription factors potentially modulate plant tolerance to low-temperature stress through the jasmonic acid (JA) pathway (Zhuang et al., 2024). In *Rosa persica*, JA signaling integrates with the ICE-CBF-COR pathway through RpMYC2, which interacts with multiple transcription factors to enhance cold tolerance (Geng et al., 2025). Additionally, cold adaptation mechanisms exhibit remarkable developmental plasticity. The R2R3-MYB member RhMYB17 in *Rosa hybrida* mediates stamen-to-petal homotic transformation by reprogramming floral organ identity genes (*RhAP2*/*RhAP2L*), thereby facilitating morphological adaptation to low-temperature environments (Yang et al., 2024). To date, little is known regarding the low-temperature response mechanisms in *R. rugosa*.

*R*. *rugosa* represents one of the most economically significant horticultural crops globally, yet cold stress severely constrains its yield and quality. This study combines transcriptomic profiling of *R*. *rugosa* under cold stress with functional characterization of core regulatory components to decipher the adaptive mechanisms to low temperatures. Our results elucidate pivotal molecular pathways and genetic determinants that underpin cold tolerance in *R*. *rugosa*, and identify actionable genetic targets for breeding cold-resistant ornamental varieties.

## 2 Materials and methods

### 2.1 Plant materials and cold treatments

Three-year-old plants of *R*. *rugosa* cultivars Zizhi and Hetian were cultivated in a controlled greenhouse (22°C/18°C day/night, 60% relative humidity, 16 h photoperiod). For cold stress treatment, uniformly grown plants were transferred to a 4°C growth chamber. Focusing primarily on Zizhi, leaf and one-year-old stem tissues were harvested from three biological replicates under control (22°C) and cold-stressed conditions (4°C for 4 h, 12 h, and 24 h) for transcriptome analysis. To specifically investigate early conserved cold responses, leaf and one-year-old stem tissues from Hetian were also harvested after 4 h of cold treatment (4°C). Immediately after collection, all tissue samples were frozen in liquid nitrogen and stored at −80°C. The sample ID was designed to consist of the cultivar abbreviation, followed by the tissue type, and then the duration of low-temperature treatment. Specifically, “Z” denotes *R*. *rugosa* Zizhi, while “H” represents *R*. *rugosa* Hetian; “L” stands for leaf tissue, and “S” indicates stem tissue.

For cold treatment experiments in transgenic *Arabidopsis*, approximately 2- to 3-week-old wild-type Col-0 and transgenic plants were exposed to −8°C for 12 h in a controlled-environment chamber, followed by a 12 h thawing phase at 4°C; subsequently, plants were transferred to standard growth conditions (23°C/16h light, 18°C/8h dark, 70,000 Lux) for a 4 day recovery period. Photographic documentation was performed immediately before cold treatment, after treatment completion, and following recovery. In parallel experiments for physiological and biochemical analyses under cold stress, similarly aged wild-type Col-0 and transgenic plants were subjected to −4°C for 12 h, after which leaf tissues were harvested, flash-frozen in liquid nitrogen, and stored at −80°C for subsequent quantification of malondialdehyde (MDA) and proline content, catalase (CAT) activity, ROS staining, and chlorophyll fluorescence imaging.

### 2.2 Library construction, sequencing, and quality assessment

Total RNA was isolated using the RNAprep Pure Plant Kit (Tiangen Biotech, Beijing, China) following the manufacturer’s protocol. RNA integrity was verified by agarose gel electrophoresis and quantified using a NanoDrop 2000 (Thermo Fisher). RNA integrity (RIN ≥ 8.0) was verified using an Agilent 2100 Bioanalyzer. Strand-specific RNA-seq libraries were constructed using the MGIEasy RNA Library Prep Kit for BGI^®^. Libraries were sequenced on the BGI high-throughput sequencing platform, DNBSEQ. Raw reads were processed with Fastp (v0.21.0) to remove adapters and low-quality sequences (Chen et al., 2018). Clean reads were aligned to the *R*. *rugosa* reference genome (GDR, https://www.rosaceae.org) (Jung et al., 2019) using Star (v2.7.9a) with default parameters (Dobin et al., 2013). Sequencing depth, Q30 scores, GC content, and mapping rates were calculated using FastQC (v0.11.9) (Davis et al., 2021). Reproducibility among biological replicates was assessed using Spearman correlation heatmaps and PCA in R (v4.2.0).

### 2.3 Differential expression and functional enrichment analysis

Differentially expressed genes (DEGs) between cold-treated and control samples (|log2FoldChange| > 1, *P*_adj_ < 0.05) were identified with DESeq2 (v1.26.0) (Love et al., 2014). Gene Ontology (GO) and Kyoto Encyclopedia of Genes and Genomes (KEGG) enrichment analyses were performed using clusterProfiler (v3.14.3) (Yu et al., 2012). Significantly enriched terms were filtered with a corrected *P* value (*P*_adj_) < 0.05. Venn diagrams were generated using the R package VennDiagram (v1.7.3). Expression trends of DEGs were visualized via hierarchical clustering in TBtools (v2.322) (Chen et al., 2020).

### 2.4 Weighted gene co-expression network analysis

A signed hybrid network was built using the WGCNA package (v1.71) in R (v4.2.2) (Langfelder and Horvath, 2008). The soft-thresholding power (β = 14) was selected based on scale-free topology (*R*² > 0.7). Modules were detected via dynamic tree cutting (minModuleSize = 60, mergeCutHeight = 0.25, deepSplit = 3). Module-trait associations were calculated using Pearson correlation between module eigengenes and cold treatment time points (0 h *vs* 4 h). Hub genes in the red module were identified based on module membership (MM > 0.9) and gene significance (GS > 0.8). All network visualizations and correlation plots were generated using the WGCNA package and ggplot2 (v3.4.2) in R (v4.2.2).

### 2.5 qRT-PCR analysis

Quantitative real-time PCR (qRT-PCR) assays were carried out to verify RNA-seq data using six genes. Total RNA extraction, quantification, and quality assessment were conducted in accordance with the methods used in our RNA-Seq library preparation. First-strand cDNA was synthesized using PrimeScript RT Master Mix (abm) following the manufacturer’s instructions. qRT-PCR was performed on an ABI QuantStudio 3 system in 20 μL reactions containing 10 μL Blastaq™ 2X qPCR MasterMix (abm), 2 μL cDNA template, 0.5 μL each of forward/reverse primers, and 7 μL nuclease-free water. The thermal cycling protocol comprised initial denaturation at 95°C for 3 min, followed by 40 cycles of 95°C for 15 s and 60°C for 1 min. *RrActin* served as the internal reference genes, with relative gene expression calculated using the comparative 2^−ΔΔCt^ method (Livak and Schmittgen, 2001). All experiments included three biological replicates, each assessed with three technical replicates. All primers were designed via NCBI Primer-BLAST, with sequences provided in **Supplementary Table 1**.

### 2.6 Genome-wide identification and characterization of CBF transcription factors in *R. rugosa*

The *R*. *rugosa* genome data was retrieved from the Genome Database for Rosaceae (GDR; https://www.rosaceae.org), and the hidden Markov model (HMM) for the AP2 domain (PF00847) was downloaded from the Pfam database (http://pfam.xfam.org). Using HMMER v3.2.1, this HMM profile was employed to search the *R*. *rugosa* protein database for AP2 domains with a stringent E-value cutoff of <1×10^−10^ (Finn et al., 2011). Concurrently, *Arabidopsis* CBF protein sequences, obtained from TAIR (https://www.arabidopsis.org), were aligned against the *R*. *rugosa* proteome using BLASTP. The resulting candidates from both HMM and BLASTP analyses were merged, and redundant genes were eliminated. Candidate proteins were subsequently validated through manual inspection of their domain architectures using SMART and Pfam, specifically selecting genes harboring the conserved amino acid sequences PKKPAGRxKFxETRHP and DSAWR flanking the AP2 domain, ultimately yielding six *R*. *rugosa* CBF family members (designated RrCBFs). Chromosomal locations of these *RrCBF* genes were mapped and visualized using MapChart software (E., 2002) based on the genome annotation, with gene nomenclature assigned according to their positional order. The conserved motifs within the RrCBF proteins were identified via MEME (https://meme-suite.org/meme/tools/meme) with the maximum number of motifs set to eight, and their distributions were visualized using TBtools (v2.322). Promoter regions, defined as the 2,000 bp sequences upstream of the translation start site (ATG) for each *RrCBF* gene, were extracted using TBtools and analyzed for *cis*-acting regulatory elements using the plantCARE database (https://bioinformatics.psb.ugent.be/webtools/plantcare/html/). Finally, the cold-responsive expression patterns of the *RrCBF* gene family members were assessed based on FPKM values derived from transcriptome data, and a heatmap was generated using TBtools for visualization.

### 2.7 Construction of *RrCBF1* and *RrCBF5* overexpression vector and genetic transformation of *Arabidopsis*

For the overexpression constructs, the complete *RrCBF1* and *RrCBF5* coding sequence (CDS) was amplified from *R*. *rugosa* cDNA. Gene-specific primers (**Supplementary Table 1**) were designed based on the CDS of *RrCBF1* and *RrCBF5* genes. PCR amplification was performed with a high-fidelity enzyme (*TransTaq*^®^ DNA Polymerase High Fidelit (HiFi)), and the resulting products were separated by 1.0% agarose gel electrophoresis. Purified fragments were ligated into the *pEASY*^®^ -T5 Zero Vector (TransGen) and transformed into *E. coli* DH5α competent cells via heat-shock. Positive transformants were screened and subjected to Sanger sequencing (Sangon Biotech, Shanghai). Sequence-verified fragments were directionally cloned into the pCAMBIA2300-35S-GFP expression vector using T4 DNA ligase. The recombinant constructs were validated by PCR amplification, restriction enzyme digestion, and DNA sequencing. Verified constructs were electroporated into *Agrobacterium tumefaciens* GV3101 competent cells and introduced into *Arabidopsis* (Col-0) via the floral dip method. Positive transgenic plants were selected on MS medium supplemented with 50 mg·L^-^¹ kanamycin and confirmed by PCR.

### 2.8 Phenotypic analysis and physiological parameter measurement of transgenic lines

Phenotypic analysis and physiological parameter measurements were conducted on wild-type Col-0 and transgenic *Arabidopsis* lines. To elucidate the biological functions of *RrCBF1* and *RrCBF5* in regulating plant growth and development, the primary phenotypic traits of *Arabidopsis* plants grown under normal conditions were photographically documented at 2, 4, and 8 weeks after cultivation. Multiple morphological traits were quantified, including the number of rosette leaves at flowering, flowering time, and rosette leaf length at 4 weeks. The number of rosette leaves, which is a well-established indicator for estimating flowering time under long-day (LD) conditions (Amasino, 2010), was recorded along with the number of days to flowering. For each trait, 10 biologically independent plants per line were examined.

The activities of CAT, as well as the contents of MDA and proline, in both wild-type and transgenic *Arabidopsis* under normal and cold stress conditions (−4°C), were determined using assay kits manufactured by Suzhou Grace Biotechnology Co., Ltd., Suzhou, China (product codes: G0106F, G0110F, and G0111F, respectively). Briefly, 0.1 g of leaf tissue was flash-frozen in liquid nitrogen and immediately homogenized using a tissue grinder. The powdered tissue was thoroughly mixed with 1 mL of the corresponding extraction solution as specified in the kit protocols. The homogenate was then centrifuged at 11,270 ×g for 10 min at 4°C, and the resulting supernatant was collected for subsequent assays. Each sample included three biological replicates.

Histochemical detection of hydrogen peroxide (H_2_O_2_) was performed using 3,3′-diaminobenzidine (DAB) staining. Leaves were immersed in a PBS solution (pH 3.8) containing 1 mg/mL DAB and incubated in darkness at 25°C overnight until brown precipitates became visible. Superoxide anion (O_2_^-^ ) was detected via nitroblue tetrazolium (NBT) staining, wherein leaves were incubated in 10 mM NBT prepared in phosphate buffer (pH 7.8) under the same dark conditions until dark blue spots developed. After staining, the solution was decanted, and chlorophyll was removed by boiling the leaves in 75% and 100% ethanol sequentially at 95°C for 15 min each. This decolorization process was repeated 1–2 times until all green pigmentation was eliminated. The samples were then photographed for documentation.

Chlorophyll fluorescence parameters were measured non-invasively using an Imaging-PAM fluorometer (Heinz Walz, Effeltrich, Germany). Both wild-type and transgenic plants were assessed before and after cold stress treatment. After dark adaptation for 30 min, the maximum quantum yield of PSII (Fv/Fm), the effective quantum yield of PSII photochemistry Y(II), as well as the quantum yield of non-regulated energy dissipation Y(NO) and quantum yield of regulated energy dissipation Y(NPQ), were recorded. For each line, 12 individual plants were measured to minimize random errors.

### 2.9 Statistical analysis

In this study, Venn diagrams, scatter plots, heatmaps, as well as GO and KEGG enrichment plots were generated using the online cloud platform (https://www.benacloud.com/#/login, BenagenCloud, Wuhan, China). Data visualization and statistical analyses were performed with GraphPad Prism (v8.0). For comparisons between two groups, Student’s *t*-test was applied, with significance levels denoted as *P < 0.05, **P < 0.01, and ***P < 0.001. Data are presented as mean ± standard deviation (SD), and a *P*-value < 0.05 was considered statistically significant.

## 3 Results

### 3.1 Quality assessment of transcriptome sequencing data

To comprehensively decipher the gene expression profiles of *R*. *rugosa* under cold stress, this study conducted transcriptome sequencing on stem and leaf tissues from *R*. *rugosa* Zizhi and Hetian under both cold-stressed and control conditions. Three biological replicates were included for each tissue-treatment combination, yielding sequencing data from 36 samples. Sequencing on the Illumina XPlus platform generated 227.96 Gb of raw data, with 218.15 Gb of high-quality clean data retained after stringent quality control, averaging 6.06 Gb per sample (**Supplementary Table 2**). All 36 libraries exhibited Q30 values ≥93% and stable GC content between 45%–46% (**Supplementary Table 2**). Clean reads mapped to the *R*. *rugosa* reference genome (Jung et al., 2019, GDR) at rates ranging from 81.74% to 90.51%, with unique mapped reads averaging 81.15% (**Supplementary Table 3**). Sample correlation heatmap and principal component analysis (PCA) confirmed high reproducibility among biological replicates (**Supplementary Figure 1**). PCA further delineated distinct clustering patterns based on cultivar (*R*. *rugosa* Zizhi *vs*. *R*. *rugosa* Hetian), tissue type (stem *vs*. leaf), and treatment condition (cold-treated *vs*. control) (**Supplementary Figure 1**). Additionally, qRT-PCR validation of six genes (including *evm.TU.Chr3.4582* and *evm.TU.Chr7.1881*) demonstrated high consistency with transcriptome sequencing data (**Supplementary Figure 2**). Collectively, these multidimensional analyses validate the high reliability and robust biological reproducibility of the transcriptome sequencing data obtained in this study.

### 3.2 Temporal dynamics of cold stress response in *R. rugosa* leaves and stems

**Figure 1:**
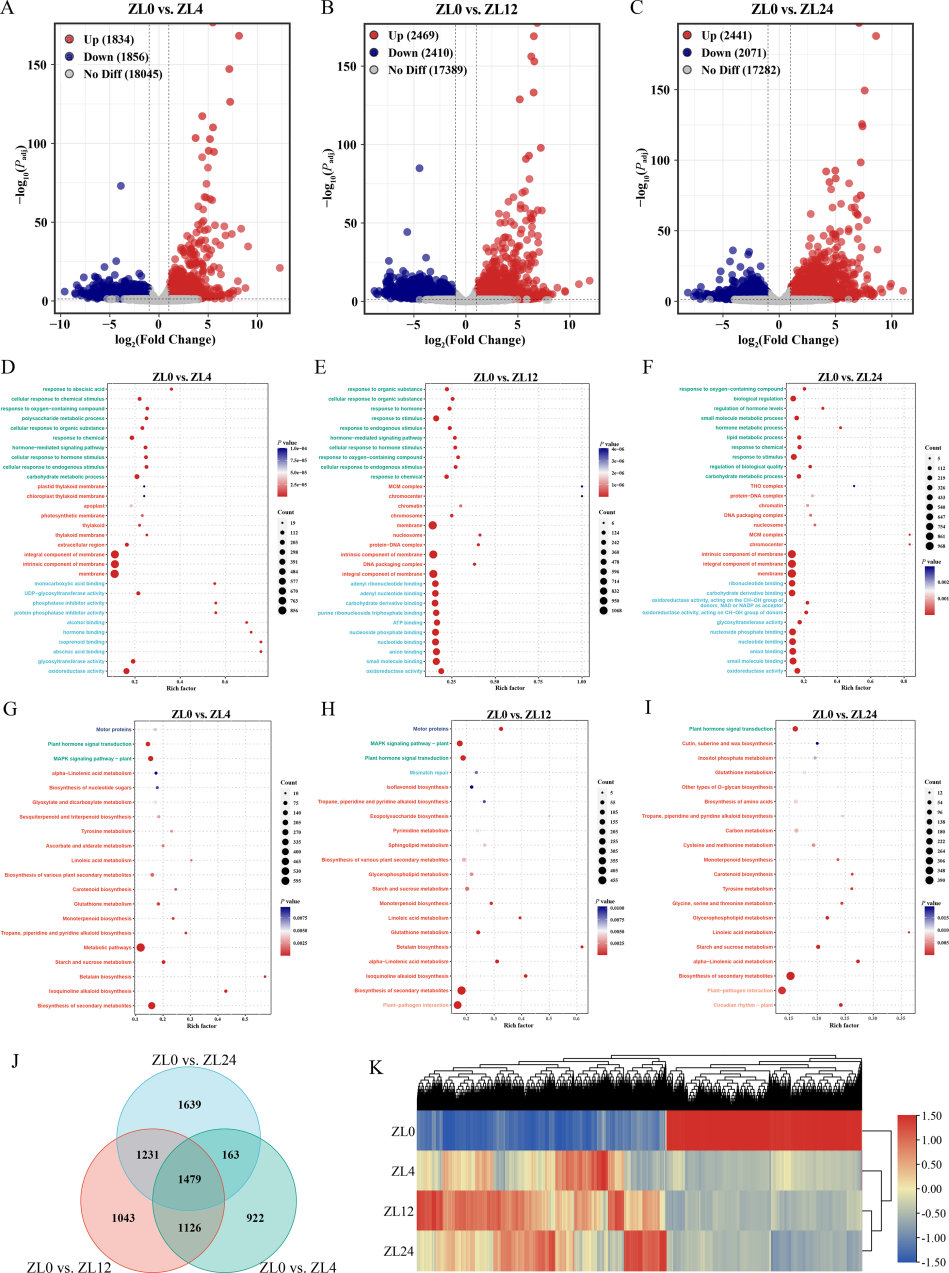
Transcriptome dynamics and functional enrichment in *R*. *rugosa* leaves under cold stress (4°C). **(A–C)** Number of DEGs in *R*. *rugosa* leaves under cold stress (4°C) at 4 h, 12 h, and 24 h compared to the 0 h control. **(D–F)** Bubble charts depicting GO functional enrichment results for the DEGs identified at 4 h, 12 h, and 24 h of cold stress. The figure shows the top ten enriched terms from each GO category: biological processes (green), cellular components (red), and molecular functions (blue). Full term lists are available in **Supplementary Table 4**-**6**. **(G–I)** Bubble charts presenting enriched KEGG pathways for DEGs at discrete cold-stress time points (4 h, 12 h, 24 h). Charts highlight top enriched pathways across major functional categories. Comprehensive pathway annotations are provided in **Supplementary Tables 7**-**9**. **(J)** Venn diagram identifying a shared set of 1,479 DEGs regulated across all cold-stress time points (4 h, 12 h, 24 h). **(K)** Hierarchical clustering heatmap displaying the expression patterns of the core 1,479 DEGs.

To investigate the molecular mechanisms underlying the response of *R*. *rugosa* leaves and stems to cold stress (4°C), we performed transcriptome sequencing on tissue samples subjected to different cold treatment durations (4 h, 12 h, 24 h), with the 0 h sample serving as the control for comprehensive comparative analysis. Differential gene expression analysis revealed that in leaves, compared to the 0 h control, 3,690 (1,834 upregulated, 1,856 downregulated), 4,879 (2,469 upregulated, 2,410 downregulated), and 4,512 (2,441 upregulated, 2,071 downregulated) DEGs were identified at 4 h, 12 h, and 24 h, respectively (**Figures 1A–C**). GO enrichment analysis of the DEGs from each time point (4 h, 12 h, 24 h *vs*. 0 h) revealed significantly enriched biological processes (BP), molecular functions (MF), and cellular components (CC) (**Figures 1D–F**). For BP, early-response genes (4 h) were significantly enriched in 118 GO terms, including carbohydrate metabolic process, cellular response to endogenous stimulus, cellular response to hormone stimulus, response to chemical, and hormone-mediated signaling pathway (**Figure 1D**, **Supplementary Table 4**). Middle-response genes (12 h) were significantly enriched in 94 GO terms, such as response to chemical, cellular response to endogenous stimulus, response to oxygen-containing compound, cellular response to hormone stimulus, and hormone-mediated signaling pathway (**Figure 1E**, **Supplementary Table 5**). Late-response genes (24 h) were significantly enriched in 59 GO terms, including carbohydrate metabolic process, regulation of biological quality, response to stimulus, response to chemical, and lipid metabolic process (**Figure 1F**, **Supplementary Table 6**). For MF, early-response genes were significantly enriched in 106 GO terms, notably oxidoreductase activity, glycosyltransferase activity, abscisic acid binding, isoprenoid binding, and hormone binding (**Figure 1D**, **Supplementary Table 4**). Middle-response genes were significantly enriched in 119 GO terms, primarily oxidoreductase activity, small molecule binding, anion binding, nucleotide binding, and nucleoside phosphate binding (**Figure 1E**, **Supplementary Table 5**). Late-response genes were significantly enriched in 60 GO terms, including oxidoreductase activity, small molecule binding, anion binding, nucleotide binding, and nucleoside phosphate binding (**Figure 1F**, **Supplementary Table 6**). For CC, early-response genes were significantly enriched in 12 GO terms, including membrane, intrinsic component of membrane, integral component of membrane, extracellular region, and thylakoid membrane (**Figure 1D**, **Supplementary Table 4**). Middle-response genes were significantly enriched in 14 GO terms, such as integral component of membrane, DNA packaging complex, intrinsic component of membrane, protein-DNA complex, and nucleosome (**Figure 1E**, **Supplementary Table 5**). Late-response genes were significantly enriched in 5 GO terms, including membrane, integral component of membrane, and intrinsic component of membrane (**Figure 1F**, **Supplementary Table 6**). Furthermore, KEGG pathway enrichment analysis identified significant enrichment of early-response genes (4 h) in 18 pathways, including biosynthesis of secondary metabolites, isoquinoline alkaloid biosynthesis, MAPK signaling pathway, betalain biosynthesis, starch and sucrose metabolism, metabolic pathways, and plant hormone signal transduction (**Figure 1G**, **Supplementary Table 7**). Middle-response genes (12 h) were significantly enriched in 16 pathways, such as biosynthesis of secondary metabolites, motor proteins, isoquinoline alkaloid biosynthesis, alpha-linolenic acid metabolism, plant-pathogen interaction, betalain biosynthesis, and plant hormone signal transduction (**Figure 1H**, **Supplementary Table 8**). Late-response genes (24 h) were significantly enriched in 12 pathways, including biosynthesis of secondary metabolites, alpha-linolenic acid metabolism, circadian rhythm, starch and sucrose metabolism, plant hormone signal transduction, glycerophospholipid metabolism, and linoleic acid metabolism (**Figure 1I**, **Supplementary Table 9**).

Additionally, Venn diagram analysis of the DEG sets identified a core set of 1,479 genes consistently differentially expressed across all three time points (4 h, 12 h, 24 h) (**Figure 1J**). This indicates that these 1,479 core genes are significantly regulated throughout the early to middle-late stages (4–24 h) of cold stress response in *R. rugosa* leaves and likely represent key components of core cold-response pathways. Notably, these core genes exhibited distinct expression patterns: 653 genes were downregulated while 826 genes were upregulated following cold stress (**Figure 1K**). The common DEGs (1,479 genes) were significantly enriched in several key pathways, including biosynthesis of secondary metabolites, starch and sucrose metabolism, carotenoid biosynthesis, linoleic acid metabolism, isoquinoline alkaloid biosynthesis, plant hormone signal transduction, circadian rhythm, and alpha-linolenic acid metabolism (**Supplementary Table 10**). Furthermore, transcriptional profiling identified 922, 1,043, and 1,639 uniquely DEGs following 4 h, 12 h, and 24 h of cold stress, respectively (**Figure 1J**, **Supplementary Figure 3**). In contrast, time-specific DEGs exhibited unique functional profiles (**Supplementary Table 10**). For the 4 h-specific DEGs (922 genes), significant enrichment was observed in photosynthesis - antenna proteins and ribosome pathways. The 12 h-specific DEGs (1,043 genes) were notably enriched in plant-pathogen interaction, motor proteins, mismatch repair, homologous recombination, and ABC transporters. Interestingly, the 24 h-specific DEGs (1,639 genes) were also enriched in plant-pathogen interaction. In summary, the cooperative response to cold stress in *R*. *rugosa* arises from the combined activities of core and stage-specific genes.

Similarly, differential gene expression analysis of cold responses in stem identified 3,444 (2,306 up-regulated, 1,138 down-regulated), 5,063 (2,902 up-regulated, 2,161 down-regulated), and 8,476 (4,615 up-regulated, 3,861 down-regulated) DEGs in the 4 h, 12 h, and 24 h cold-treated groups, respectively, compared to the 0 h control (**Supplementary Figures 4A–C**). GO enrichment analysis of DEGs from these time points (4 h, 12 h, 24 h *vs* 0 h) revealed significantly enriched BP, MF, and CC (**Supplementary Figures 4D–F**). For BP, early-response (4 h) genes were significantly enriched in 91 GO terms including carbohydrate metabolic process, polysaccharide metabolic process, microtubule-based movement, microtubule-based process, and response to chemical (**Supplementary Figure 4D**, **Supplementary Table 11**); middle-response (12 h) genes in 74 terms including carbohydrate metabolic process, phenylpropanoid metabolic process, phenylpropanoid catabolic process, lignin catabolic process, and lignin metabolic process (**Supplementary Figure 4E**, **Supplementary Table 12**); and late-response (24 h) genes in 146 terms including carbohydrate metabolic process, carbohydrate biosynthetic process, polysaccharide metabolic process, cellular carbohydrate metabolic process, and polysaccharide biosynthetic process (**Supplementary Figure 4F**, **Supplementary Table 13**). For MF, early-response genes showed significant enrichment in 67 terms including microtubule binding, tubulin binding, hydrolase activity, cytoskeletal motor activity, and cytoskeletal protein binding (**Supplementary Figure 4D**, **Supplementary Table 11**); middle-response genes in 73 terms including small molecule binding, nucleotide binding, nucleoside phosphate binding, oxidoreductase activity, and oxidoreductase activity (**Supplementary Figure 4E**, **Supplementary Table 12**); and late-response genes in 109 terms including small molecule binding, nucleotide binding, nucleoside phosphate binding, anion binding, and carbohydrate derivative binding (**Supplementary Figure 4F**, **Supplementary Table 13**). For CC, early-response genes were enriched in 14 terms including extracellular region, apoplast, supramolecular polymer, supramolecular fiber, and polymeric cytoskeletal fiber (**Supplementary Figure 4D**, **Supplementary Table 11**); middle-response genes in 6 terms including apoplast, integral component of membrane, intrinsic component of membrane, membrane, and extracellular region (**Supplementary Figure 4E**, **Supplementary Table 12**); and late-response genes in 40 terms including integral component of membrane, intrinsic component of membrane, cytoplasm, Golgi membrane, and Golgi apparatus (**Supplementary Figure 4F**, **Supplementary Table 13**). Furthermore, KEGG pathway enrichment analysis revealed that genes responsive at the early stage (4 h) were significantly enriched in 10 pathways, including motor proteins, MAPK signaling pathway, plant hormone signal transduction, starch and sucrose metabolism, amino sugar and nucleotide sugar metabolism, sulfur metabolism, and pentose and glucuronate interconversions (**Supplementary Figure 4G**, **Supplementary Table 14**). Genes showing mid-term responses (12 h) exhibited significant enrichment in 9 pathways, such as motor proteins, biosynthesis of secondary metabolites, starch and sucrose metabolism, glycine, serine and threonine metabolism, circadian rhythm, sphingolipid metabolism, and MAPK signaling pathway (**Supplementary Figure 4H**, **Supplementary Table 15**). Finally, genes with late-stage responses (24 h) were significantly enriched in 15 pathways, including starch and sucrose metabolism, motor proteins, glycine, serine and threonine metabolism, amino sugar and nucleotide sugar metabolism, biosynthesis of secondary metabolites, biosynthesis of nucleotide sugars, and carbon metabolism (**Supplementary Figure 4I**, **Supplementary Table 16**).

Venn diagram analysis further uncovered a core set of 1,872 persistently DEGs common to all three time points (**Supplementary Figure 4J**), indicating their significant regulation throughout early to middle-late cold stress and potential involvement in core response pathways. Notably, the expression patterns of these core genes distinctly clustered into two groups: 616 genes were down-regulated while 1,256 genes were up-regulated following cold stress (**Supplementary Figure 4K**). KEGG enrichment analysis of the common DEGs revealed significant enrichment in pathways related to motor proteins and starch and sucrose metabolism (**Supplementary Table 17**). To delineate the temporal specificity of the transcriptional response to cold stress (4°C) in stem tissues, we profiled uniquely DEGs across three distinct time points. Transcriptional profiling revealed 789, 681, and 4,017 DEGs that were uniquely identified following 4 h, 12 h, and 24 h of cold stress, respectively (**Supplementary Figure 4J**, **Supplementary Figure 5**). In contrast, time-specific DEGs had distinct functions (**Supplementary Table 17**). For the 4 h-specific DEGs (789 genes), significant enrichment was observed in plant hormone signal transduction, plant-pathogen interaction, carbon fixation by Calvin cycle, MAPK signaling pathway, and motor proteins. The 12 h-specific DEGs (681 genes) were notably enriched in biosynthesis of secondary metabolites and sesquiterpenoid and triterpenoid biosynthesis. The 24 h-specific DEGs (4,017 genes) were enriched in photosynthesis - antenna proteins and phagosome pathways. In summary, the collective response to cold stress in stem tissues is shaped by sustained core transcriptional programs alongside discrete stage-specific mechanisms.

### 3.3 Core transcriptional responses to early cold stress (4 h) revealed by intersection analysis in two *R. rugosa* cultivars

**Figure 2:**
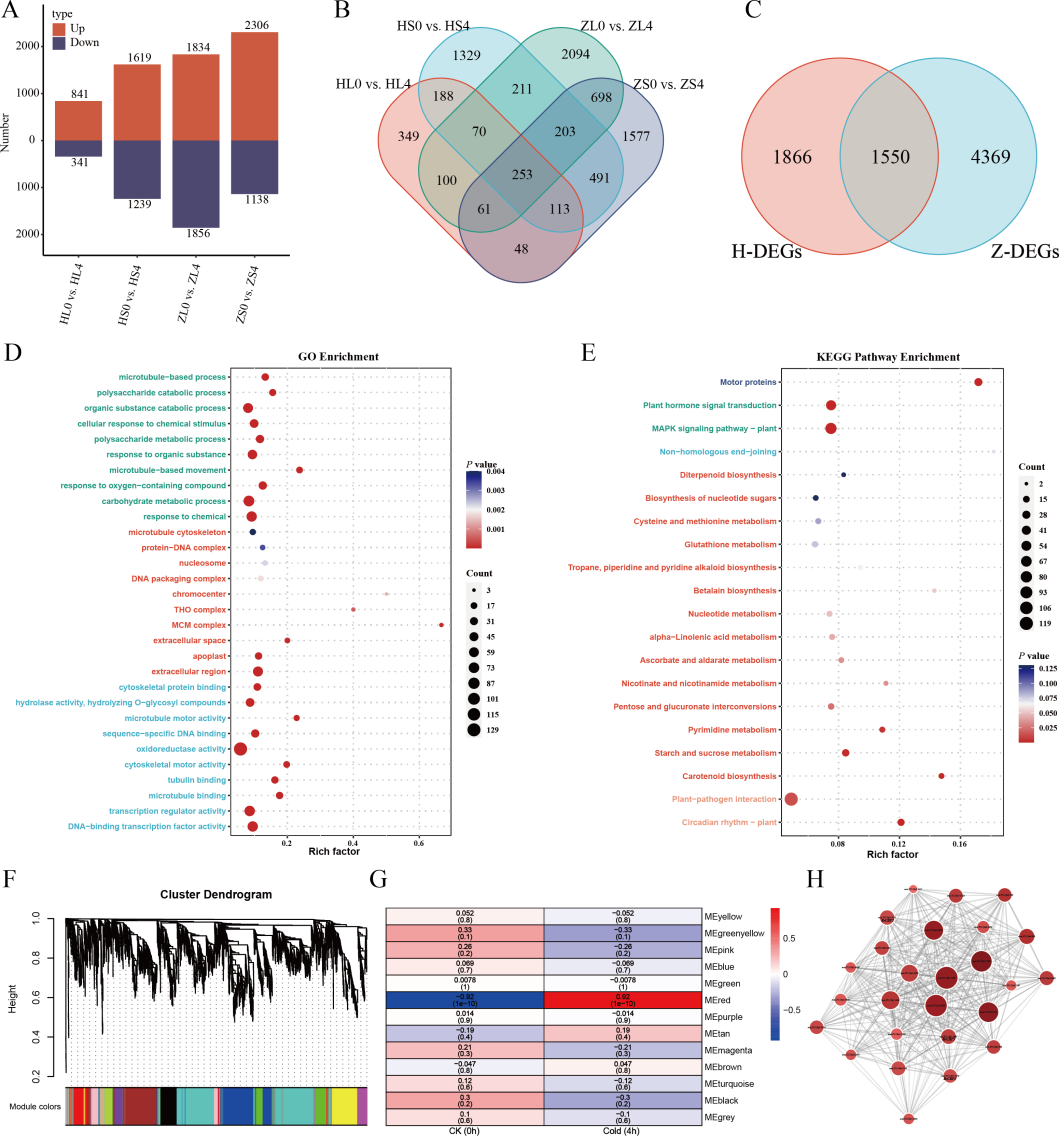
Integrated transcriptomic analysis of conserved early cold responses (4 h) in two *R*. *rugosa* cultivars. **(A)** Number of DEGs in stems and leaves of *R*. *rugosa* Zizhi and Hetian after 4 h cold stress. Upregulated (red) and downregulated (blue) DEGs are shown. **(B)** Overlapping DEGs between cultivars in stem and leaf tissues. **(C)** Core conserved cold-responsive DEGs shared across both cultivars. **(D)** GO enrichment analysis of core conserved cold-responsive DEGs. Presented are the top ten enriched GO terms, categorized into biological processes (green), cellular components (red), and molecular functions (blue). **(E)** KEGG pathway enrichment of core conserved cold-responsive DEGs. **(F)** Hierarchical clustering dendrogram of co-expression modules. **(G)** Module-trait associations correlating gene modules with cold stress (4 h at 4°C). **(H)** Gene co-expression network of the MEred module.

To elucidate the core biological processes underlying the early cold response (4 h) in *R*. *rugosa*, we conducted transcriptome analyses on both leaves and stems of *R*. *rugosa* Zizhi and Hetian subjected to 4 h cold treatment, followed by identification of DEGs relative to the 0 h controls. Comparative analysis revealed a total of 5,919 DEGs in Zizhi under cold stress, comprising 2,306 upregulated and 1,138 downregulated genes in stems, and 1,834 upregulated and 1,856 downregulated genes in leaves. Similarly, 3,416 DEGs were identified in Hetian, with 1,619 upregulated and 1,239 downregulated genes in stems, and 841 upregulated and 341 downregulated genes in leaves (**Figure 2A**). Further investigation demonstrated that the two cultivars shared 1,060 DEGs in stem tissues and 484 DEGs in leaf tissues (**Figure 2B**). Intersection analysis of all DEGs across both cultivars ultimately identified 1,550 common DEGs, representing conserved early cold-responsive genes in *R*. *rugosa* (**Figure 2C**).

GO and KEGG enrichment analyses were performed on the 1,550 relatively conserved early cold-responsive genes identified above. GO enrichment analysis identified a total of 67 significantly enriched terms in the BP category, among which the most representative were response to chemical, carbohydrate metabolic process, response to oxygen-containing compound, microtubule-based movement, and response to organic substance (**Figure 2D**, **Supplementary Table 18**). In the CC category, four terms were significantly enriched, mainly associated with the extracellular region, apoplast, extracellular space, and MCM complex (**Figure 2D**, **Supplementary Table 18**). For MF, 32 terms exhibited significant enrichment, with notable involvements in DNA-binding transcription factor activity, transcription regulator activity, microtubule binding, tubulin binding, and cytoskeletal motor activity (**Figure 2D**, **Supplementary Table 18**). KEGG pathway enrichment analysis revealed significant enrichment in six key pathways, including motor proteins, the MAPK signaling pathway, plant hormone signal transduction, circadian rhythm, carotenoid biosynthesis, as well as starch and sucrose metabolism (**Figure 2E**, **Supplementary Table 19**). These integrated GO and KEGG analyses collectively demonstrate that the early cold response is characterized by multifaceted regulatory mechanisms, encompassing signal transduction, transcriptional regulation, cytoskeleton-driven dynamics, and metabolic reprogramming, thereby elucidating the coordinated molecular strategies that underpin early cold response in *R*. *rugosa*.

Subsequently, we performed WGCNA on *R*. *rugosa* Hetian and Zizhi subjected to cold stress at 0 h and 4 h. Using the FPKM values, a total of 13 distinct co-expression modules were constructed by WGCNA and designated with different colors (**Figure 2F**). In these modules, the MEred was the most significantly correlated with cold treatment, showing a strong positive association (*r* = 0.92, *p* = 1e-10) with the 4 h time point (**Figure 2G**). Within this key module, 26 high-connectivity hub genes were discerned based on stringent criteria (MM > 0.9 and GS > 0.8) (**Figure 2H**). Notably, three CBF transcription factors (evm.model.Chr7.1871, evm.model.Chr7.1876, and evm.model.Chr7.1881) were identified among these hub genes. The high connectivity of these *RrCBF* genes within the co-expression network suggest their potential role as key regulators in the transcriptional reprogramming associated with cold stress response, highlighting their critical function in the molecular mechanism underlying cold tolerance in *R*. *rugosa*.

### 3.4 The *RrCBF* gene family as a putative central regulator in *R. rugosa* cold stress response

**Figure 3:**
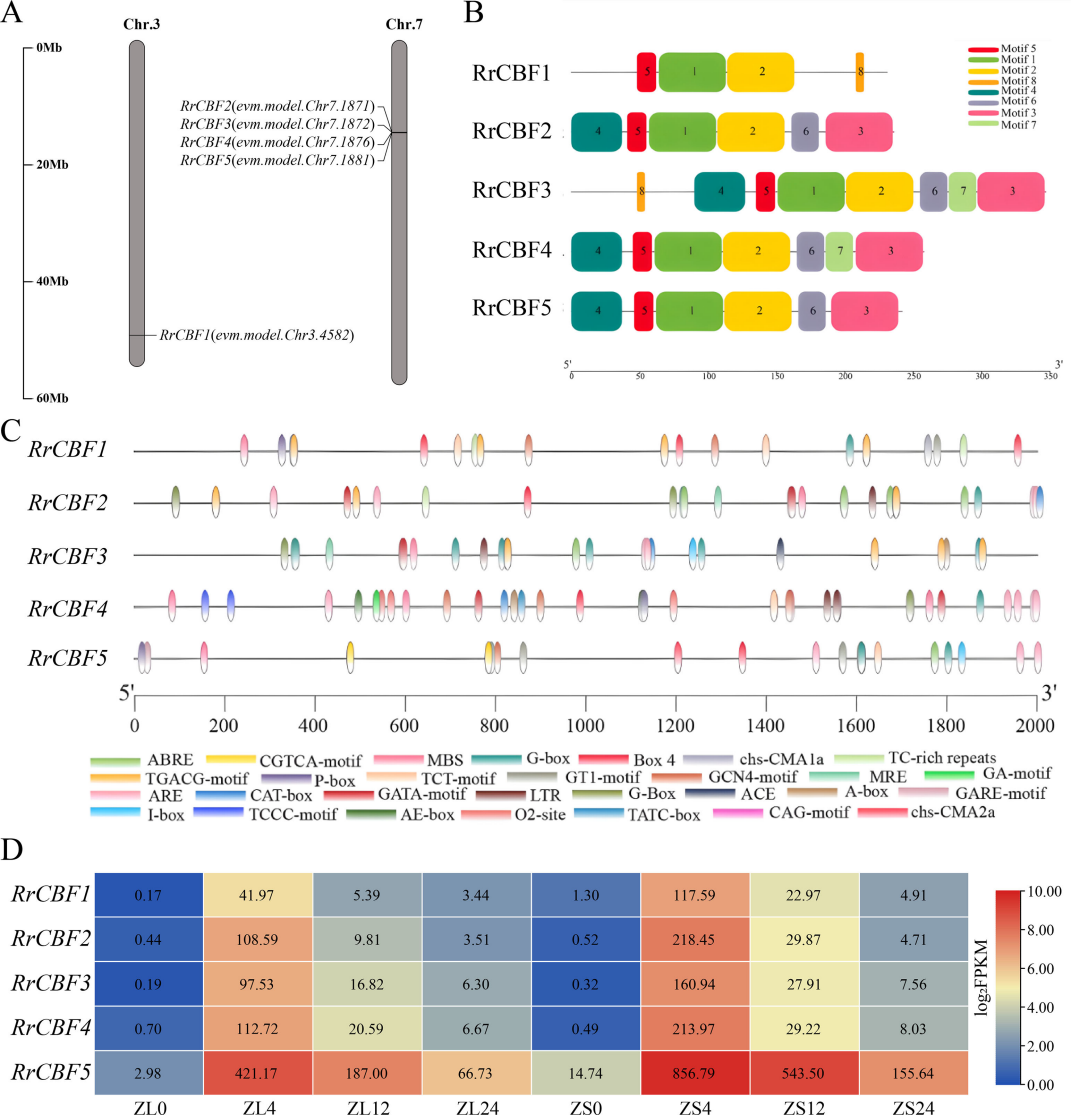
Genome-wide identification and expression analysis of the RrCBF transcription factor family in *R*. *rugosa*. **(A)** Chromosomal distribution of the five identified *RrCBF* genes (*RrCBF1*–*RrCBF5*). **(B)** Conserved motif composition within the *RrCBF* proteins. **(C)** In silico identification of putative cis-acting regulatory elements within the 2 kb promoter regions upstream of the *RrCBF* gene. **(D)** Spatiotemporal expression profiles of *RrCBF* genes under cold stress (4°C). Heatmap depicts log_2_FPKM values derived from transcriptome data.

Based on the WGCNA described above, *RrCBF* was identified as a potential key regulator in the cold response of *R*. *rugosa*. Consequently, we performed a genome-wide identification and characterization of the *RrCBF* gene family in *R*. *rugosa* and analyzed their expression patterns under cold stress. Gene family analysis revealed five *R*. *rugosa RrCBF* genes, designated as *RrCBF1* to *RrCBF5* according to their chromosomal locations (**Figure 3A**). The gene IDs for *RrCBF1* to *RrCBF5* are as follows: *evm.model.Chr3.4582* (*RrCBF1*), *evm.model.Chr7.1871* (*RrCBF2*), *evm.model.Chr7.1872* (*RrCBF3*), *evm.model.Chr7.1876* (*RrCBF4*), and *evm.model.Chr7.1881* (*RrCBF5*). These five members were distributed across two chromosomes: *RrCBF1* was located on chromosome 3, while *RrCBF2* to *RrCBF5* formed a cluster on chromosome 7 (**Figure 3A**). Analysis of conserved motifs within the RrCBF protein family identified eight conserved motifs (**Figure 3B**). Motifs 1, 2, and 5 were highly conserved among the RrCBF proteins, being present in all five members with consistent arrangement. Some motifs exhibited gene specificity: Motifs 3, 4, and 6 were present in four RrCBF proteins, while Motifs 7 and 8 were found in only two (**Figure 3B**). An analysis of cis-acting elements was performed on the 2,000 bp promoter sequences upstream of the *RrCBFs*. This investigation identified the presence of various elements implicated in responses to abiotic stress (TC-rich repeats), drought (MBS), low temperature (LTR), methyl jasmonate (CGTCA-motif, TGACG-motif), gibberellin (P-box, TATC-box, GARE-motif), and abscisic acid (ABRE), among others (**Figure 3C**). Utilizing FPKM values derived from *R*. *rugosa* transcriptome data, we analyzed the expression patterns of the five *RrCBF* members in different tissues (leaves and stems) across four cold treatment time points (**Figure 3D**). The results demonstrated that the expression levels of all five *RrCBF* genes significantly increased with prolonged cold stress duration, peaking at 4 h. This indicates that all five *RrCBF* genes are induced by low temperature. Notably, expression levels were higher in stems compared to leaves, and began to show varying degrees of downregulation at 12 h (**Figure 3D**). Among the five *RrCBF* genes, the promoter regions of *RrCBF2*, *RrCBF3*, and *RrCBF4* contained the low-temperature-responsive cis-acting element LTR. In contrast, no LTR element was identified in the promoters of *RrCBF1* and *RrCBF5* (**Figure 3C**). Nevertheless, all five *RrCBF* genes were induced by cold stress (**Figure 3D**). qRT-PCR analysis confirmed the cold-induced expression of *RrCBF1* and *RrCBF5* (**Supplementary Figure 2**). However, the absence of the LTR element in their promoters raises questions about their specific role in the low-temperature response of *R*. *rugosa*, warranting further functional investigation.

### 3.5 *RrCBF1/RrCBF5* overexpression alters developmental timing and morphology in *Arabidopsis*

**Figure 4:**
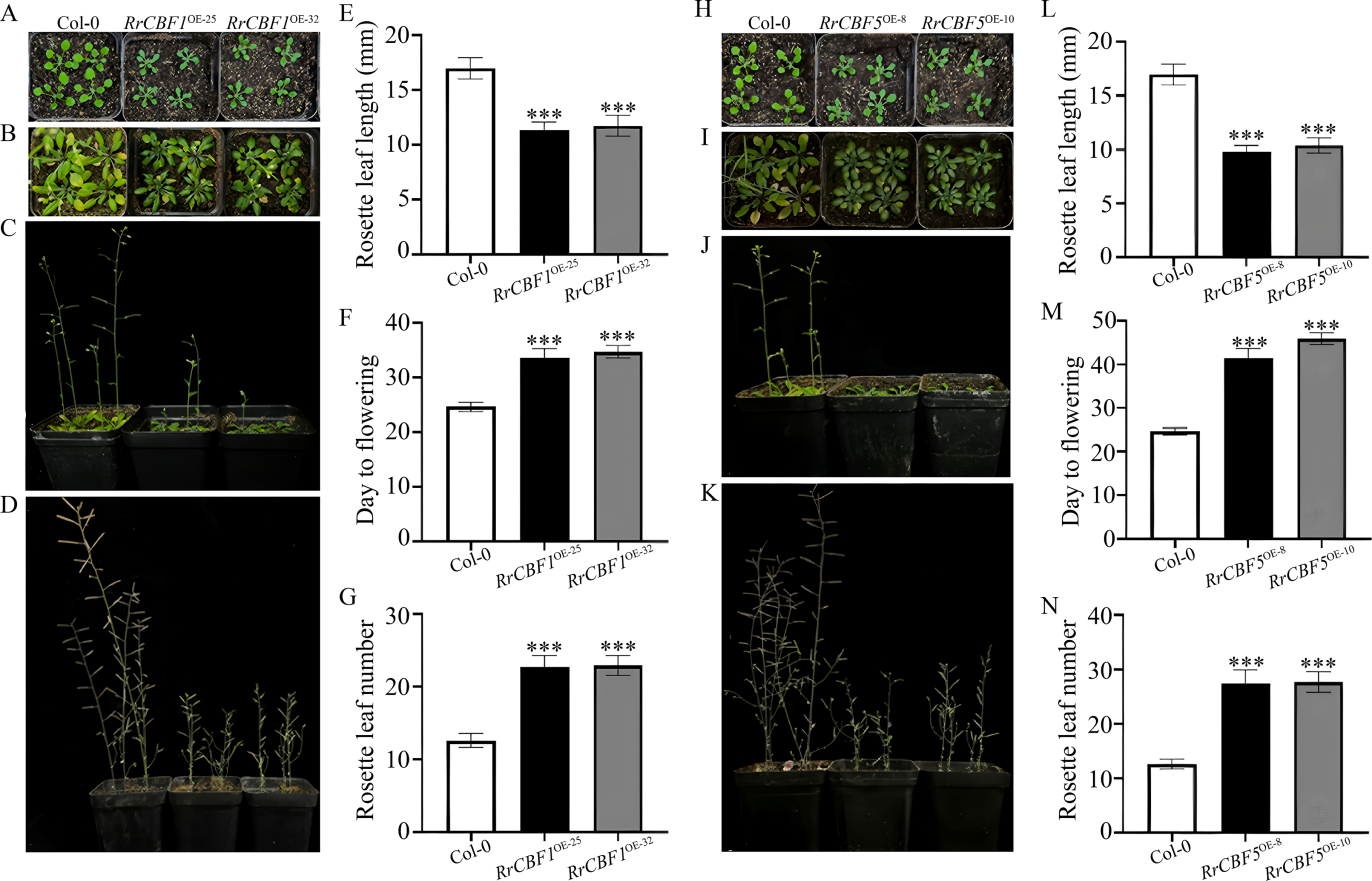
Phenotypic characterization of wild-type (Col-0 ecotype) and transgenic *Arabidopsis* overexpressing *RrCBF1* or *RrCBF5*. **(A, H)** Representative phenotypes of 2-week-old soil-grown seedlings overexpressing *RrCBF1***(A)** and *RrCBF5***(H)**. **(B, C, I, J)** Representative phenotypes of 4-week-old soil-grown seedlings overexpressing *RrCBF1***(B, C)** and *RrCBF5***(I, J)**, shown in top **(B, I)** and front **(C, J)** views. **(D, K)** Whole-plant phenotypes at 8 weeks for *RrCBF1***(D)** and *RrCBF5***(K)**. **(E, L)** Rosette leaf length quantification at 4 weeks for *RrCBF1***(E)** and *RrCBF5***(L)**. **(F, M)** Flowering time for *RrCBF1***(F)** and *RrCBF5***(M)**. **(G, N)** Rosette leaf number at flowering in *RrCBF1***(G)** and *RrCBF5***(N)**. Data are presented as mean ± SD (*n* = 10 biologically independent plants). Asterisks denote statistically significant differences compared to the wild-type (Col-0), as determined by a two-tailed Student’s *t*-test (***P < 0.001).

To further elucidate the functions of *RrCBF1* and *RrCBF5*, both genes were cloned and sequenced. The CDS obtained were 696 bp for *RrCBF1* and 729 bp for *RrCBF5*. The target fragments were subsequently cloned into the pCAMBIA2300-35S-GFP overexpression vector and heterologously transformed into *Arabidopsis* via the floral dip method for functional characterization. Phenotypic analysis comparing wild-type Col-0 with *RrCBF1*- or *RrCBF5*-transgenic *Arabidopsis* lines revealed significant developmental alterations in both transgenic lines. At the seedling stage, transgenic plants (*RrCBF1* and *RrCBF5*) exhibited markedly reduced stature compared to wild-type Col-0, accompanied by smaller, thicker leaves with intensified green pigmentation (**Figures 4A–D, H–K**). Regarding leaf development, rosette leaves of 4-week-old wild-type Col-0 measured 15.08 to 18.61 mm in length. In contrast, rosette leaf lengths in *RrCBF1* and *RrCBF5* transgenic lines were significantly shorter, ranging from 10.21 to 13.26 mm and 9.04 to 11.12 mm, respectively, demonstrating highly significant differences from the wild-type (**Figures 4E, L**). Significant alterations in reproductive growth regulation were also observed. Wild-type Col-0 plants flowered within 23 to 26 days after germination, producing 11 to 14 rosette leaves at the time of flowering. In contrast, flowering was significantly delayed in both *RrCBF1* and *RrCBF5* transgenic lines, occurring at 31–36 days and 38–47 days, respectively (**Figures 4F, M**). Concurrently, the number of rosette leaves significantly increased to 21–26 and 24–32 (**Figures 4G, N**). These findings indicate a prolonged vegetative growth phase and a significantly delayed transition to reproductive growth in plants overexpressing either *RrCBF1* or *RrCBF5* (*P* < 0.05). Collectively, the phenotypic data demonstrate that overexpression of *RrCBF1* or *RrCBF5* results in dwarfism, reduced leaf size, increased leaf thickness, darker green leaves, delayed growth and flowering, and a significant suppression of the transition from vegetative to reproductive growth in *Arabidopsis*, with *RrCBF5* exhibiting more pronounced regulatory effects.

### 3.6 Heterologous overexpression of *RrCBF1* and *RrCBF5* in *Arabidopsis* enhances cold tolerance through regulation of antioxidant and photosynthetic pathways

**Figure 5:**
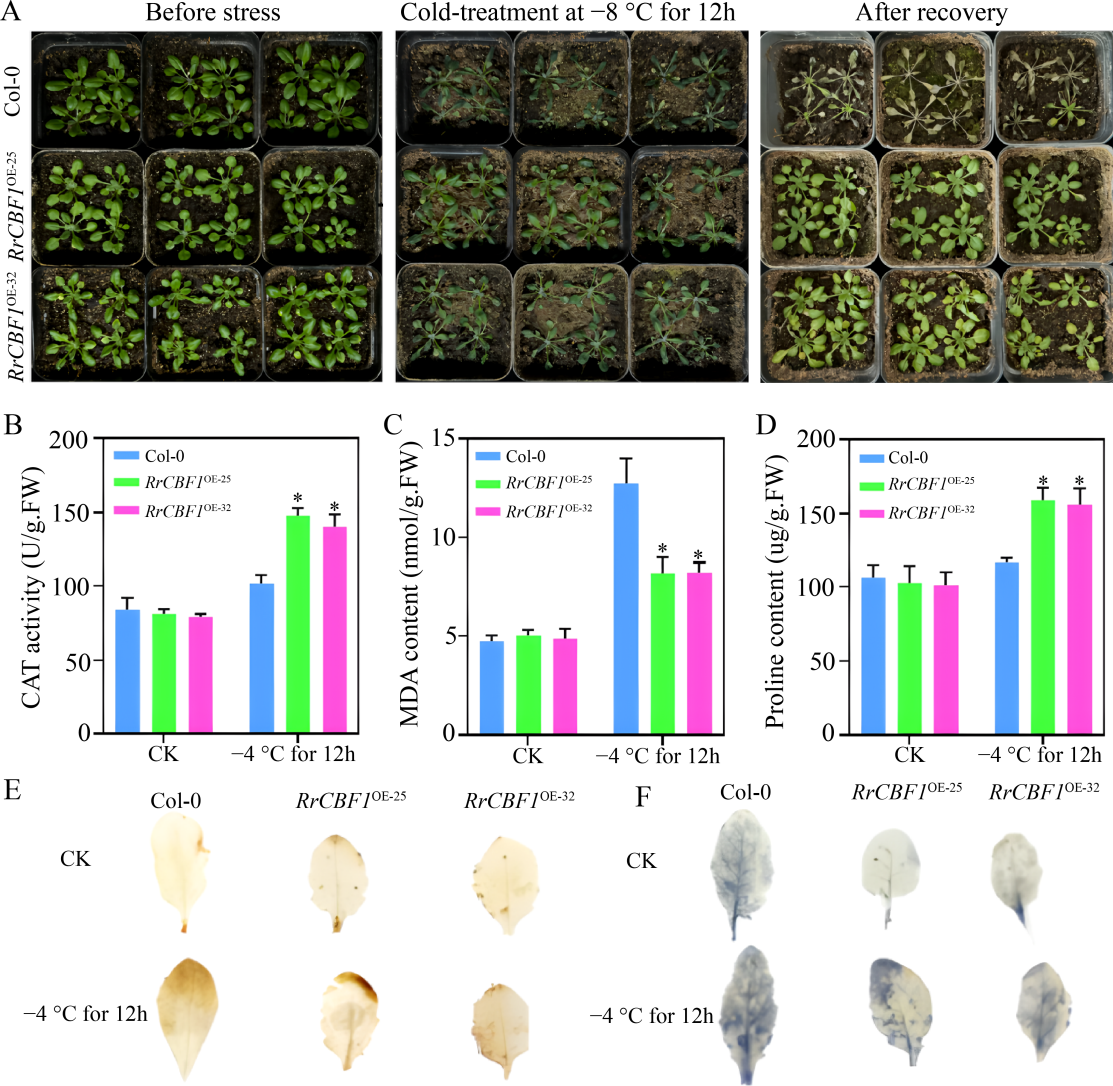
Heterologous overexpression of *RrCBF1* enhances cold tolerance in *Arabidopsis*. **(A)** Phenotypic responses of wild-type (Col-0) and *35S:RrCBF1* transgenic lines after 12 h exposure to −8°C and following 4-day recovery. **(B–D)** Levels of MDA **(B)**, CAT activity **(C)**, and proline content **(D)** in Col-0 and *RrCBF1*-overexpressing plants under normal conditions (22°C) and after 12 h at −4°C. Values are mean ± SD of three replications. Significant differences relative to the Col-0 are indicated by asterisks (*P < 0.05, two-tailed Student’s *t*-test). **(E–F)** Histochemical detection of H_2_O_2_ (DAB staining, E) and O_2_^-^ (NBT staining, F) accumulation in leaves after cold stress.

**Figure 6:**
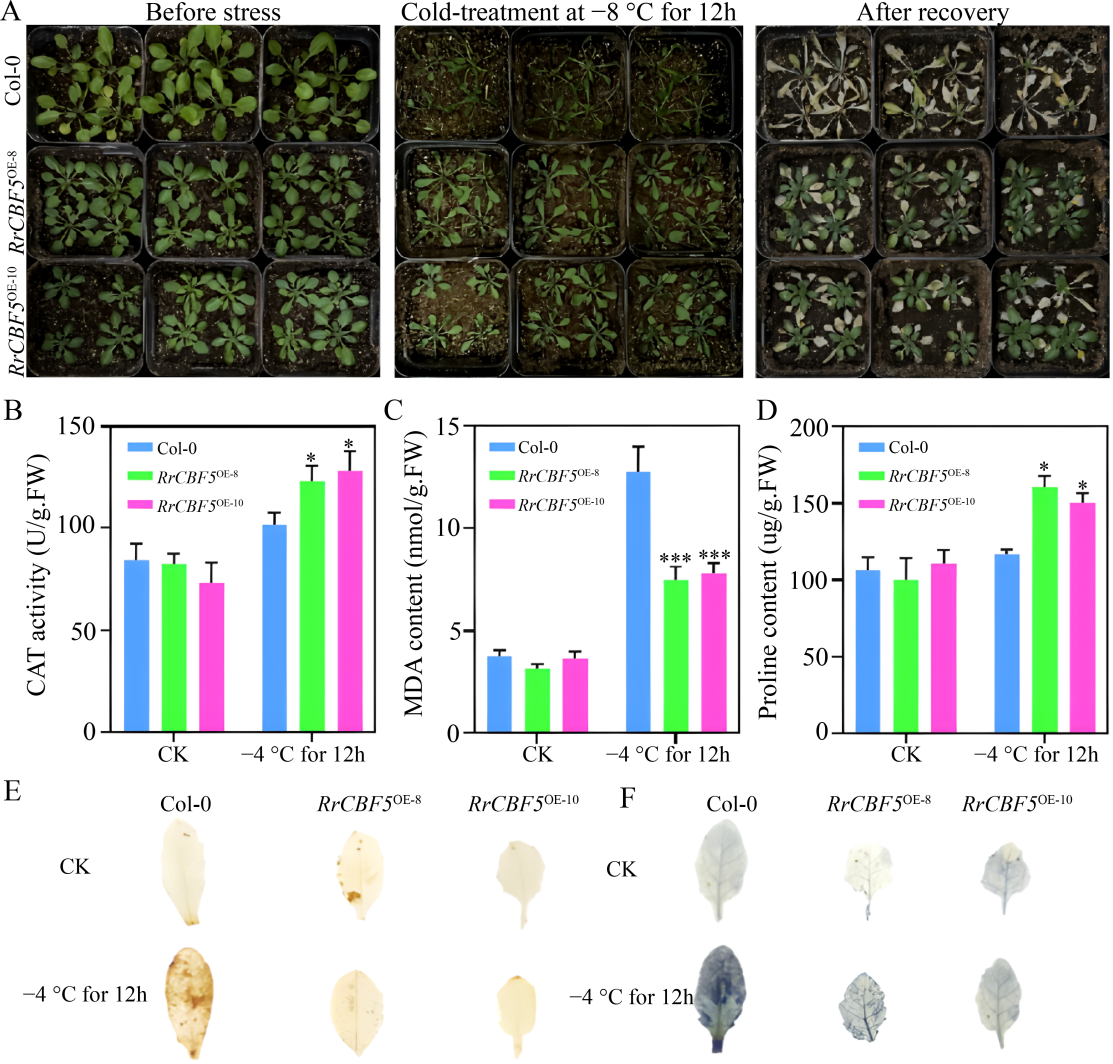
*RrCBF5* overexpression confers improved cold resistance in *Arabidopsis*. **(A)** Comparison of phenotypes between wild-type (Col-0) and *35S:RrCBF5* transgenic lines subjected to −8°C for 12 h followed by 4 days of recovery. **(B–D)** Measurements of MDA **(B)**, CAT activity **(C)**, and proline content **(D)** in the wild-type (Col-0) and *RrCBF5*-overexpressing plants at 22°C and after 12 h at −4°C. Values are mean ± SD of three replications. Asterisks denote statistically significant differences from the Col-0, as assessed by a two-tailed Student’s *t*-test (*P < 0.05, ***P < 0.001). **(E, F)** Histochemical visualization of H_2_O_2_**(E)** and O_2_^-^**(F)** accumulation in leaves following cold stress.

To investigate the potential involvement of *R*. *rugosa RrCBF1* and *RrCBF5* in freezing tolerance, we subjected *Arabidopsis* plants overexpressing *RrCBF1* or *RrCBF5* to subzero temperatures. Following a 12 h exposure to −8°C, wild-type Col-0 plants exhibited severe leaf damage, characterized by extensive wilting and shrinkage (**Figures 5A**, **6A**). In contrast, transgenic plants expressing either *RrCBF1* or *RrCBF5* displayed only localized leaf injury, with the majority of leaves retaining normal morphology (**Figures 5A**, **6A**). After four days of recovery under standard growth conditions, transgenic plants demonstrated significant regrowth, whereas wild-type Col-0 leaves were completely bleached, exhibiting a substantially lower survival rate compared to the transgenic lines (**Figure 5A**, **6A**). These distinct phenotypic responses clearly indicate that overexpression of *RrCBF1* or *RrCBF5* enhances freezing tolerance in *Arabidopsis*.

Subsequently, we analyzed osmotic adjustment and the antioxidant defense system, known mechanisms associated with improved freezing tolerance, in plants overexpressing *RrCBF1* or *RrCBF5*. Under normal temperature conditions, no significant differences (*P* > 0.05) were observed in MDA content, CAT activity, or proline content between wild-type Col-0 and transgenic plants (**Figures 5B–D**, **6B–D**). However, after a 12 h cold treatment at −4°C, the transgenic lines exhibited significantly higher CAT activity and proline content (*P* < 0.05), along with significantly lower MDA levels (*P* < 0.05), compared to wild-type Col-0 (**Figures 5B–D**, **6B–D**). Histochemical staining with DAB and NBT revealed elevated H_2_O_2_ and O_2_^-^ accumulation in both wild-type and transgenic plants under cold stress; nevertheless, the accumulation was markedly lower in the transgenic lines than in wild-type Col-0 (**Figures 5E, F**, **6E, F**). These results demonstrate that the enhanced freezing tolerance conferred by heterologous overexpression of *RrCBF1* or *RrCBF5* is attributable to reduced lipid peroxidation and membrane damage, coupled with increased accumulation of osmolytes, thereby improving resistance to freezing injury.

Furthermore, photosynthetic characteristics were examined in both wild-type and transgenic lines (**Supplementary Figure 6**). Under normal growth conditions, Fv/Fm and Y(NPQ) showed no significant differences between wild-type Col-0 and plants overexpressing *RrCBF1* or *RrCBF5* (**Supplementary Figures 6A, D, E, H**). However, Y(NO) was significantly higher in transgenic plants expressing either gene compared to the wild type (**Supplementary Figures 6C, G**). Additionally, Y(II) was significantly higher in *RrCBF5*-overexpressing plants than in Col-0 (**Supplementary Figure 6F**). Following a 12 h cold treatment at −4°C, transgenic plants exhibited significantly higher values for Fv/Fm, Y(II), and Y(NPQ) (*P* < 0.05), along with a significantly lower Y(NO), compared to wild-type Col-0 (**Supplementary Figures 6A–H**). These findings indicate that both *RrCBF1* and *RrCBF5* genes mitigate chilling-induced photoinhibition by enhancing the stability of PSII and its photoprotective capacity.

## 4 Discussion

The response of *R*. *rugosa* to 4°C low-temperature stress is a complex and dynamic process involving the synergistic actions of signal transduction, metabolic reprogramming, and defense mechanisms. Leaves and one-year-old stems exhibit both shared strategies and distinct organ-specific adaptations during cold acclimation. This response follows a clear temporal trajectory, transitioning from rapid signal perception and early stress reactions to mid- to long-term metabolic adjustments and homeostasis restoration. Within the first 4 h of cold exposure, *R*. *rugosa* rapidly activates MAPK cascades and plant hormone signaling pathways, which likely collaborate to induce the expression of stress-responsive genes to mitigate chilling injury (Liu and Zhou, 2018; Qian et al., 2024; Li et al., 2025a). Concurrently, the upregulated expression of motor proteins may facilitate intracellular substance redistribution and cellular structural reorganization, alleviating cold-induced dysfunctions. Metabolically, *R*. *rugosa* initiate starch and sucrose metabolism, converting starch into soluble sugars to supply energy and enhance osmotic protection. By 12 h, the cold response becomes more systematic, with sustained upregulation of MAPK signaling pathway, motor protein activity, and starch and sucrose metabolism pathways. Furthermore, significant enrichment was observed in sphingolipid metabolism, which has been reported to be crucial for maintaining membrane fluidity and function, thereby enhancing cold tolerance in plants (Huang et al., 2025). Additionally, the increased biosynthesis of secondary metabolites, such as flavonoids, enhances cold stress adaptation by enhancing antioxidant capacity, stabilizing cell membranes, and modulating the expression of stress-responsive genes (Li et al., 2025b). After 24 h, *R*. *rugosa* enter a phase of systemic adaptation characterized by continued activation of starch/sucrose metabolism and secondary metabolite biosynthesis, along with the induction of glycine, serine, and threonine metabolism. This latter pathway supports one-carbon metabolism, facilitating DNA/RNA repair and glutathione synthesis to further strengthen antioxidant defenses. Collectively, *R*. *rugosa* employs a multi-dimensional coordination strategy spanning rapid sensing, metabolic reorganization, and long-term defense adaptation to achieve a holistic cold response. Notably, key pathways including MAPK signaling, plant hormone signal transduction, starch/sucrose metabolism, and motor protein-mediated structural dynamics are consistently engaged in both leaves and stems, underscoring their roles as essential functional modules in initial signal conversion, energy supply, and cellular restructuring under cold stress.

The response strategies of *R*. *rugosa* leaves and stems to cold stress exhibit remarkable organ specificity. As the primary organs for perception and defense, leaves adopt a more proactive and diverse approach, primarily through extensive synthesis of protective compounds such as antioxidants and osmoregulators to directly counteract stress. In contrast, the stems, serving as structural and conductive organs, employ more conservative and fundamental strategies focused on ensuring energy and basic material supply. Specifically, in leaves, alterations in glycerophospholipid metabolism contribute to maintaining membrane fluidity and stability while acting as signaling molecules, thereby enhancing cold tolerance (Wu et al., 2022). The synthesis pathways of secondary metabolites in leaves under cold stress are notably diverse, encompassing alkaloids, terpenoids, phenolics, as well as glutathione and ascorbate metabolism, highlighting a frontline defense strategy centered on producing antioxidants, osmoprotectants, and defensive compounds. In comparison, stems exhibit fewer types of secondary metabolites but show significant enrichment in carbon metabolism and glycolysis/gluconeogenesis, indicating enhanced carbohydrate catabolism to supply energy and carbon skeletons. Concurrently, enrichments in amino sugar and nucleotide sugar metabolism, pentose and glucuronate interconversions, and ABC transporters suggest stems play a critical role in modulating sugar composition and enhancing transmembrane transport capacity to facilitate resource and energy redistribution throughout the plant. Collectively, leaves and stems achieve systemic synergistic cold resistance through shared core pathways involving signal transduction, antioxidant defense, and basal metabolism. However, functional divergence leads to distinct strategies—leaves prioritize active defense by modulating lipid composition, enhancing antioxidant capacity, and synthesizing protective metabolites, while stems emphasize structural maintenance and resource allocation by strengthening central carbon metabolism and sugar transport capacity, thus forming an integrated and functionally complementary system.

To precisely delineate the early mechanisms underlying cold response in *R*. *rugosa*, we focused on transcriptomic alterations at an early stage of cold stress (4 h). To distill the most central and conserved mechanisms from complex differential expressions, we intersected transcriptomic changes in stem and leaf tissues from two *R*. *rugosa* cultivars (Zizhi and Hetian) subjected to early cold stress (4 h), thereby mitigating cultivar-specific background noise and highlighting core biological processes underlying cold response. Through comparative analysis of transcriptomic profiles, we identified a core set of 1,550 cold-responsive genes (**Figure 2C**) that were consistently differentially expressed in both cultivars, suggesting their potential role in representing conserved regulatory mechanisms during early cold stress adaptation in *R*. *rugosa*. Our integrated GO and KEGG analyses of the early cold-responsive transcriptome reveal a coordinated multi-level regulatory program underpinning cold adaptation in *R*. *rugosa*. Signaling cascades were rapidly activated, as evidenced by the significant enrichment of related terms, including the MAPK signaling pathway and plant hormone signal transduction. Concurrently, the prominence of DNA-binding transcription factor activity and transcriptional regulator activity reflects a rapid reprogramming of gene expression. Furthermore, cytoskeleton-related processes, including microtubule-based movement and motor activity, suggest active reorganization of cellular architecture. Metabolic adjustments are also evident, through enriched pathways in carbohydrate metabolism, starch and sucrose metabolism, and carotenoid biosynthesis. Collectively, these findings delineate an early cold response strategy characterized by the interplay between transcriptional control, cytoskeletal dynamics, signal transduction, and metabolic reorganization, providing a systemic molecular framework for early cold-responsive in *R*. *rugosa*. Numerous homologs of well-documented cold-responsive transcription factors were encompassed within this core gene set, including *CBF3* (Shi et al., 2017), *CBF4* (Haake et al., 2002), *DDF1* (Kang et al., 2011), and *ERF105* (Bolt et al., 2017). Particularly critical was the significant enrichment of plant hormone signaling signal transduction and MAPK signaling pathways in the KEGG analysis, underscoring the pivotal roles of plant hormone signaling signals and MAPK cascades in early cold perception and signal transduction. Additionally, the activation of starch and sucrose metabolism pathways indicated that *R*. *rugosa* may rapidly adjust carbon metabolism strategies to provide energy and osmotic regulators in response to low temperatures. Using WGCNA, we identified a gene module (MEred) that exhibited the strongest correlation with the 4 h cold treatment. Within this module, 26 highly connected hub genes were discerned (**Figures 2G, H**). Among these hub genes, three encode CBF transcription factors (evm.model.Chr7.1871, evm.model.Chr7.1876, and evm.model.Chr7.1881). As central regulators in the plant cold acclimation pathway, CBF transcription factors are known to mediate low-temperature signal transduction and activate multiple downstream cold-tolerance genes (Shi et al., 2018; Nie et al., 2022). Collectively, these findings reveal key mechanisms through which *R*. *rugosa* transcriptionally coordinate the activation of an early cold-defense system.

WGCNA analysis suggested that RrCBF may serve as a key regulator in the cold response of *R*. *rugosa*. In this study, five CBF transcription factor members (*RrCBF1*–*5*) were identified in the *R*. *rugosa* genome. Among them, *RrCBF2*–*RrCBF5* form a tandem gene cluster on chromosome 7. This clustering pattern is consistent with the conservation of *CBF* gene clusters observed in other plant species such as *Arabidopsis thaliana*, *Liriodendron chinense* (Guan et al., 2021), and *Prunus persica* (Artlip et al., 2013), suggesting potential functional redundancy or cooperative regulation among *CBF* genes in plant cold stress signaling pathways. Analysis of cis-acting elements in the promoter regions of *RrCBFs* identified various regulatory motifs. All five *RrCBFs* contain MBS, ABRE, and G-box motifs, with the G-box being the most prevalent. This indicates that their expression may be induced by ABA, various stresses, and developmental processes (Fujita et al., 2005; Wang et al., 2025). Additionally, developmental regulatory elements such as the endosperm expression-related motif (GCN4 motif) (Onodera et al., 2001) and gibberellin responsiveness elements (P-box, TATC-box, GARE-motif) (Ding et al., 2024) were detected, implying that *RrCBFs* may function not only in abiotic stress responses but also in growth and developmental regulation. Notably, although a typical cold response element (LTR) was identified only in the promoters of *RrCBF2*, *RrCBF3*, and *RrCBF4*, cold-induced expression analysis demonstrated that all five *RrCBF* genes were significantly upregulated in both leaves and stems under cold stress, with expression peaks occurring at 4 h followed by a decline (**Figure 3D**). This result suggests that the cold-induced expression of *RrCBF1* and *RrCBF5* may not depend on the canonical LTR element, but rather involve alternative regulatory mechanisms. For instance, in pepper, the bHLH transcription factor CabHLH035 can regulate the expression of *CaCBF1A* by binding to the G-box element in its promoter (Zhang et al., 2024b), indicating that elements such as the G-box in *RrCBFs* may mediate cold induction under certain conditions. The putative cold-responsive elements identified in the *RrCBF* promoters through bioinformatics remain to be functionally validated as cis-regulatory motifs. To elucidate the precise roles of these motifs in mediating cold-induced *RrCBF* expression, further investigation employing promoter-reporter assays is warranted to assess the transcriptional activity of *RrCBF* under cold stress. Additionally, transcription factors interacting with the *RrCBF* promoter regions should be systematically identified via integrated bioinformatic prediction and yeast one-hybrid screening, followed by validation of direct binding between candidate transcription factors and potential cis-elements such as the G-box using chromatin immunoprecipitation quantitative PCR (ChIP-qPCR). Addressing these questions in subsequent studies will not only consolidate our understanding of RrCBF regulatory mechanisms but also contribute to a more comprehensive model of the transcriptional network underlying cold acclimation in *R*. *rugosa*.

This study demonstrates that heterologous expression of *RrCBF1* and *RrCBF5* from *R*. *rugosa* significantly enhances cold tolerance in *Arabidopsis* through coordinated regulation of multiple physiological mechanisms. *RrCBF1* and *RrCBF5* mediate the activation of antioxidant defense and osmotic adjustment pathways, which collectively contribute to the maintenance of membrane integrity. This protective effect is evidenced by diminished MDA levels and reduced ROS accumulation, ultimately promoting enhanced cellular homeostasis under cold stress (−4°C). Furthermore, the stabilization of PSII and reinforcement of photoprotective mechanisms mediated by *RrCBF1* and *RrCBF5* alleviated photoinhibition, thereby enhancing cold tolerance and maintaining photosynthetic efficiency under cold stress (−4°C). Heterologous expression of *RrCBF1* and *RrCBF5* from *R*. *rugosa* in *Arabidopsis* significantly enhanced cold tolerance in the transgenic plants, indicating that the function of *CBF* genes in cold tolerance is relatively conserved between *R*. *rugosa* and *Arabidopsis*. *CBFs*/*DREB1s* genes have been identified as originating from ancient angiosperms, with independent expansions during periods of global cooling in different eudicots (Nie et al., 2022). Thus, we speculate that the functional conservation of *CBF* genes between *R*. *rugosa* in *Arabidopsis* may be attributed to their shared ancestry in ancient angiosperms. However, as a perennial woody plant, *R*. *rugosa* may have evolved distinct mechanisms of cold adaptation compared to the herbaceous model plant *Arabidopsis*. In *Arabidopsis*, CBF1, CBF2, and CBF3 regulate 1,945, 2,482, and 1,267 target genes, respectively. These target genes are extensively involved in modulating *COR* gene expression, early cold signaling transduction, lipid and sugar metabolism, as well as hormone and environmental signal pathways (Song et al., 2021). The diversity of CBF target genes suggests that although the core function of *CBF* in cold tolerance is conserved, its downstream regulatory networks may differ substantially between these two plant types. In contrast to *Arabidopsis*, the stems of *R*. *rugosa* are perennially exposed to fluctuating environmental conditions, and their cold tolerance mechanisms are likely attributable primarily to the mechanical strength and structural composition of the stems. For instance, Eucalyptus species develop thicker and more heavily lignified xylem cell walls under cold stress (Ployet et al., 2018). Based on this, we hypothesize that the downstream network of RrCBFs may be preferentially enriched in pathways related to lignin biosynthesis, cell wall thickening, and modification. However, these hypotheses require further experimental validation, such as identifying direct target genes of RrCBFs via ChIP-seq, to elucidate whether RrCBFs regulate specific pathways in woody plants that are distinct from those in herbaceous species.

Notably, overexpression of *RrCBF1* and *RrCBF5* induced phenotypes including prolonged vegetative growth (delayed flowering), dwarfism, and smaller leaves in *Arabidopsis*, consistent with previous reports in other plant species (Gilmour et al., 2004; Park et al., 2015), suggesting a conserved role for *CBF* genes in the growth-stress trade-off and potentially serving as a key molecular basis for winter survival strategies in perennial *R*. *rugosa*. The constitutive expression of *CBF* retards plant growth, which may be associated with its regulation of gibberellin metabolism-mediated accumulation of growth-inhibiting DELLA proteins (Achard et al., 2008). Integrating expression analysis of *RrCBFs* under cold stress with transgenic functional studies, this work identifies *RrCBF1* and *RrCBF5* as key cold responsive transcription factors in *R*. *rugosa*; their heterologous expression confers significantly enhanced tolerance to cold stress in *Arabidopsis*, manifested as higher survival rates, improved growth status, and substantial improvements in the aforementioned key physiological indices.

## 5 Conclusions

This study comprehensively deciphers the molecular mechanisms of cold stress response in *R*. *rugosa* through multi-tissue and multi-temporal transcriptome profiling. We generated high‐quality transcriptome data that robustly captured spatiotemporal expression dynamics in leaves and stems under cold stress. Differential gene expression and enrichment analyses revealed a phased response involving rapid signal transduction, metabolic reprogramming, and antioxidant defense, with key roles played by MAPK signaling, plant hormone signal transduction, starch and sucrose metabolism, and biosynthesis of secondary metabolites. A core set of conserved early-response genes was identified, and WGCNA nominated *RrCBFs* as central regulators. Further genome-wide characterization identified five *RrCBF* genes, all cold-induced. Functional studies demonstrated that heterologous overexpression of *RrCBF1* or *RrCBF5* in *Arabidopsis* conferred enhanced freezing tolerance by boosting antioxidant capacity, osmotic adjustment, and photosystem protection, albeit at the cost of delayed development. Our findings provide a valuable resource and critical insights into the molecular basis of cold adaptation in *R*. *rugosa*, with potential applications in the genetic improvement of cold tolerance in *R*. *rugosa* and other woody plants.

## Data Availability

The datasets presented in this study can be found in online repositories. The names of the repository/repositories and accession number(s) can be found below: https://www.ncbi.nlm.nih.gov/, PRJNA1348705.

## Supplementary material

Supplementary Figure 1 Quality assessment of transcriptome sequencing data across 36 samples.

Supplementary Figure 2 Correlation analysis between qRT-PCR and RNA-seq data for six selected genes.

Supplementary Figure 3 Hierarchical clustering of time point-specific DEGs in *R*. *rugosa* leaves under 4°C cold stress.

Supplementary Figure 4 Transcriptome dynamics and functional enrichment in *R*. *rugosa* one-year-old stem under cold stress (4°C).

Supplementary Figure 5 Hierarchical clustering of time point-specific DEGs in *R*. *rugosa* one-year-old stem under cold stress (4°C).

Supplementary Figure 6 Chlorophyll fluorescence in WT and *RrCBF1*/*RrCBF5*-OE *Arabidopsis* under control (22°C) and cold stress (−4°C).

Supplementary Table 1 Primer sequences used in this study.

Supplementary Table 2 Transcriptome sequencing data quality analysis.

Supplementary Table 3 Summary of mapping status for RNA-seq data.

Supplementary Table 4 GO enrichment analysis of DEGs in the ZL0 *vs* ZL4 comparison.

Supplementary Table 5 GO enrichment analysis of DEGs in the ZL0 *vs* ZL12 comparison.

Supplementary Table 6 GO enrichment analysis of DEGs in the ZL0 *vs* ZL24 comparison.

Supplementary Table 7 KEGG enrichment analysis of DEGs in the ZL0 *vs* ZL4 comparison.

Supplementary Table 8 KEGG enrichment analysis of DEGs in the ZL0 *vs* ZL12 comparison.

Supplementary Table 9 KEGG enrichment analysis of DEGs in the ZL0 *vs* ZL24 comparison.

Supplementary Table 10 KEGG enrichment analysis of the common and specific DEGs in ZL under cold stress across time points.

Supplementary Table 11 GO enrichment analysis of DEGs in the ZS0 *vs* ZS4 comparison.

Supplementary Table 12 GO enrichment analysis of DEGs in the ZS0 *vs* ZS12 comparison.

Supplementary Table 13 GO enrichment analysis of DEGs in the ZS0 *vs* ZS24 comparison.

Supplementary Table 14 KEGG enrichment analysis of DEGs in the ZS0 *vs* ZS4 comparison.

Supplementary Table 15 KEGG enrichment analysis of DEGs in the ZS0 *vs* ZS12 comparison.

Supplementary Table 16 KEGG enrichment analysis of DEGs in the ZS0 *vs* ZS24 comparison.

Supplementary Table 17 KEGG enrichment analysis of the common and specific DEGs in ZS under cold stress across time points.

Supplementary Table 18 GO enrichment analysis of 1550 core conserved cold-responsive DEGs.

Supplementary Table 19 KEGG enrichment analysis of 1550 core conserved cold-responsive DEGs.
