# Supplementary material for: Transcriptome sequencing and functional analysis reveal RrCBF genes as key regulators of cold adaptation in Rosa rugosa
Source: Front Plant Sci. 2025 Dec 10;16:1732552. doi: 10.3389/fpls.2025.1732552 (PMC12728433; doi:10.3389/fpls.2025.1732552)
Supplement: Supplementary Figure 1 — Quality assessment of transcriptome sequencing data across 36 samples. [file SupplementaryFile1.docx]

Supplementary Material

Supplementary Figures

**
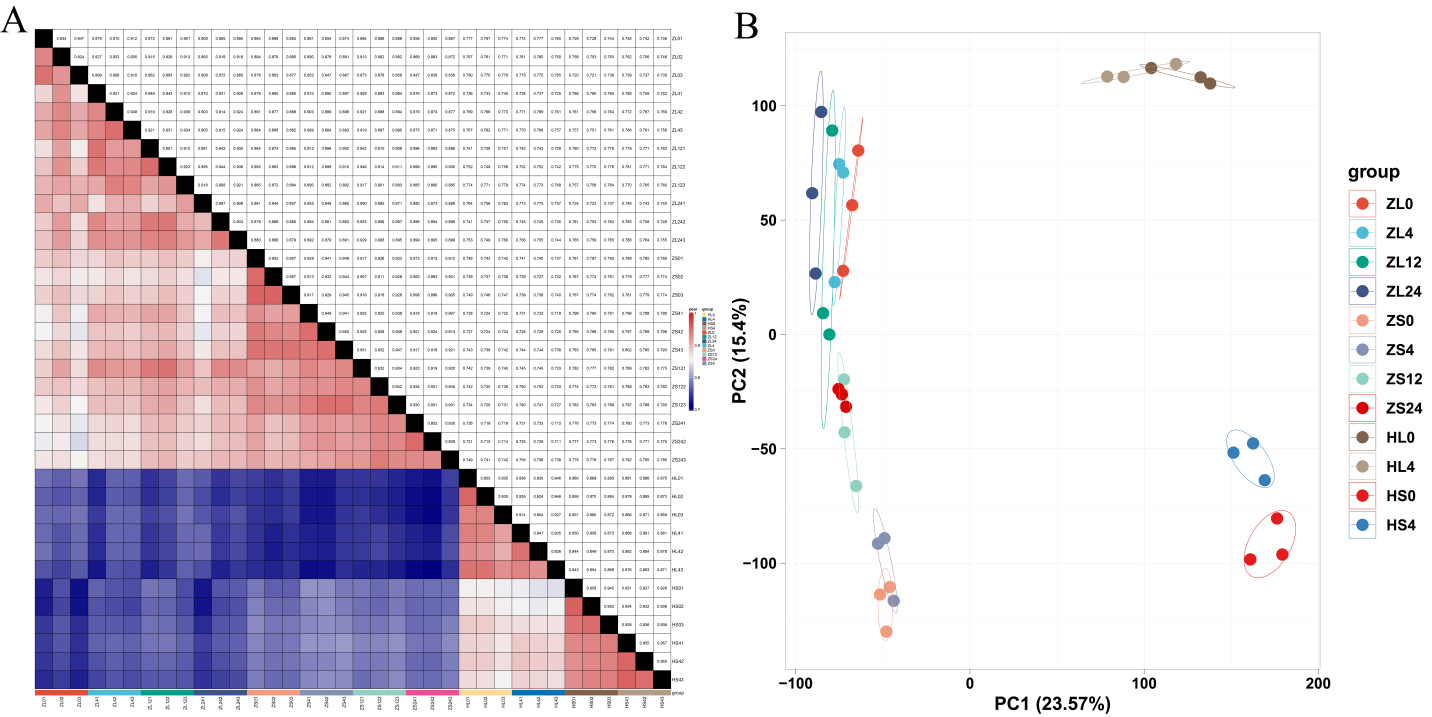
**

**Supplementary Figure 1.** Quality assessment of transcriptome sequencing data across 36 samples.

(A) Inter-sample Spearman correlation matrix. The heatmap depicts pairwise correlation coefficients of gene expression profiles between all 36 samples, indicating high reproducibility among biological replicates. (B) Principal component analysis (PCA) plot. The projection of samples in the two-dimensional space defined by the first two principal components (PC1 and PC2) illustrates the overall transcriptome variance and clustering pattern among samples.


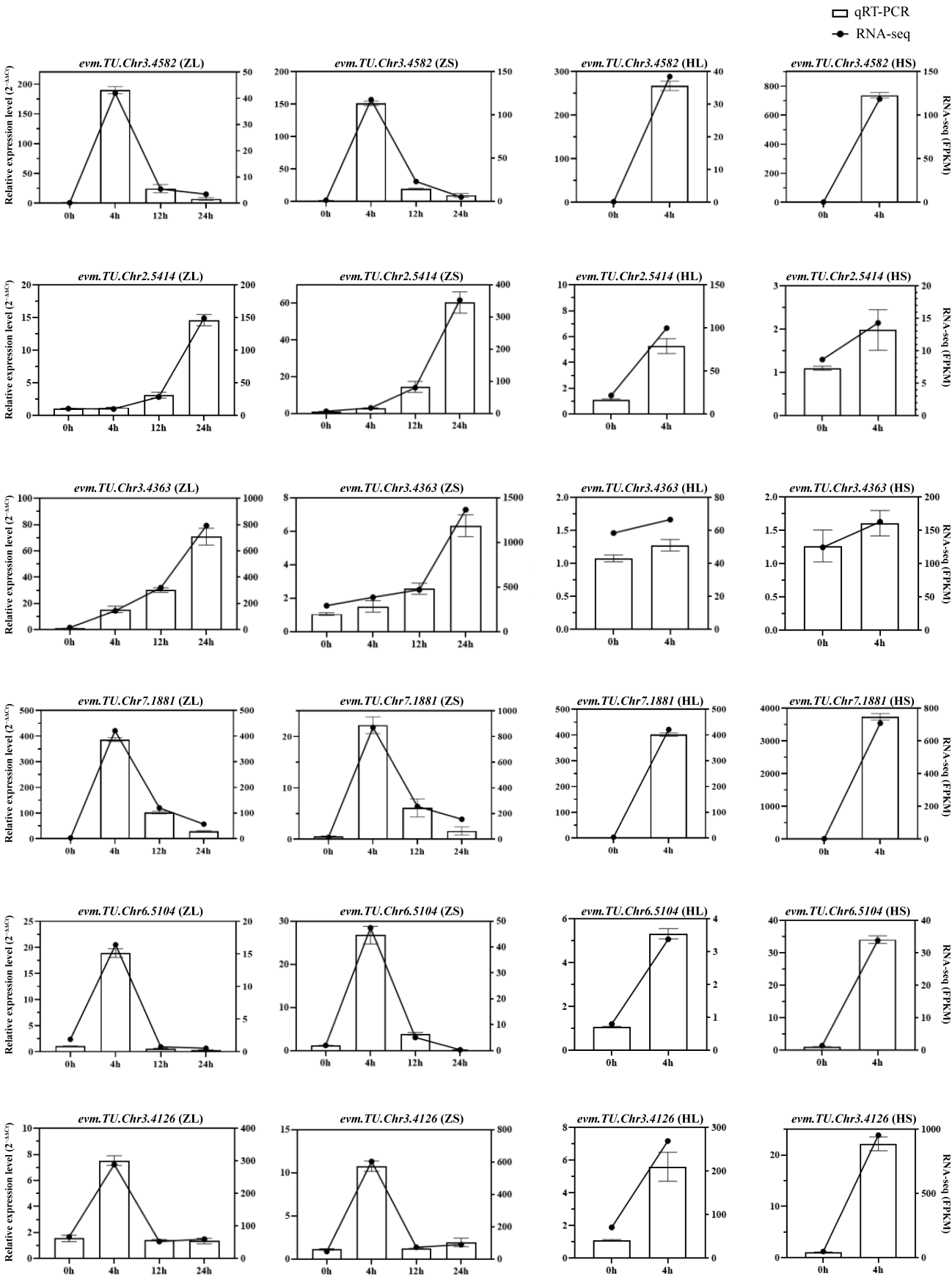


**Supplementary Figure 2.** Correlation analysis between qRT-PCR and RNA-seq data for six selected genes.

The expression levels of six genes were measured by qRT-PCR and compared with their FPKM values from the RNA-seq data. Specifically, “Z” denotes R. rugosa Zizhi, while “H” represents R. rugosa Hetian; “L” stands for leaf tissue, and “S” indicates stem tissue. All qRT-PCR primers sequences are listed in Supplementary Table 1.


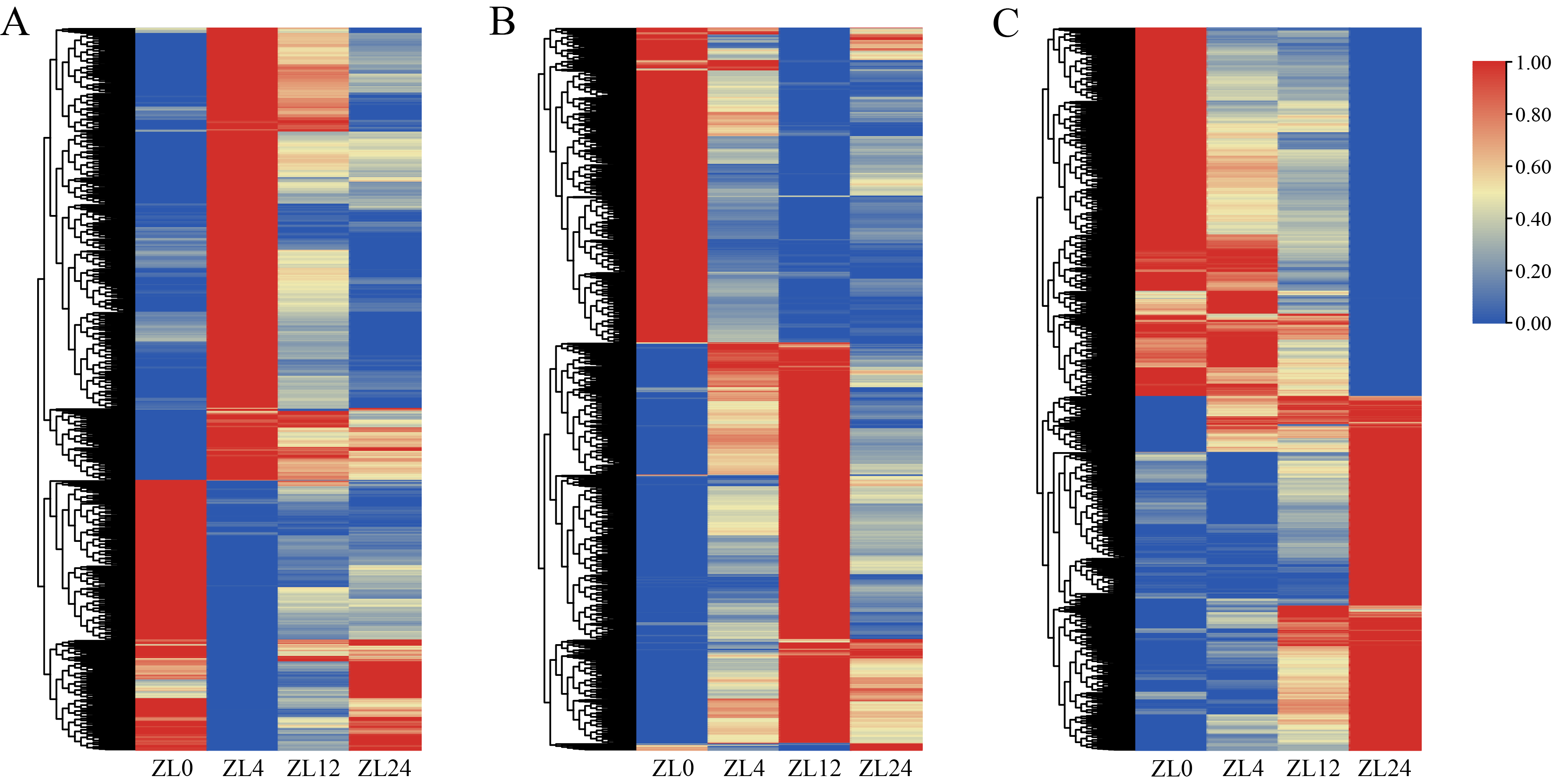


**Supplementary Figure 3.** Hierarchical clustering of time point-specific DEGs in *R*. *rugosa* leaves under 4°C cold stress.

(A) Hierarchical clustering heatmap of 922 DEGs specifically identified in leaves after 4 h of cold stress (4°C). (B) Hierarchical clustering heatmap of 1,043 DEGs unique to the 12 h cold stress. (C) Hierarchical clustering heatmap of 1,639 DEGs specifically induced by 24 h of cold stress.


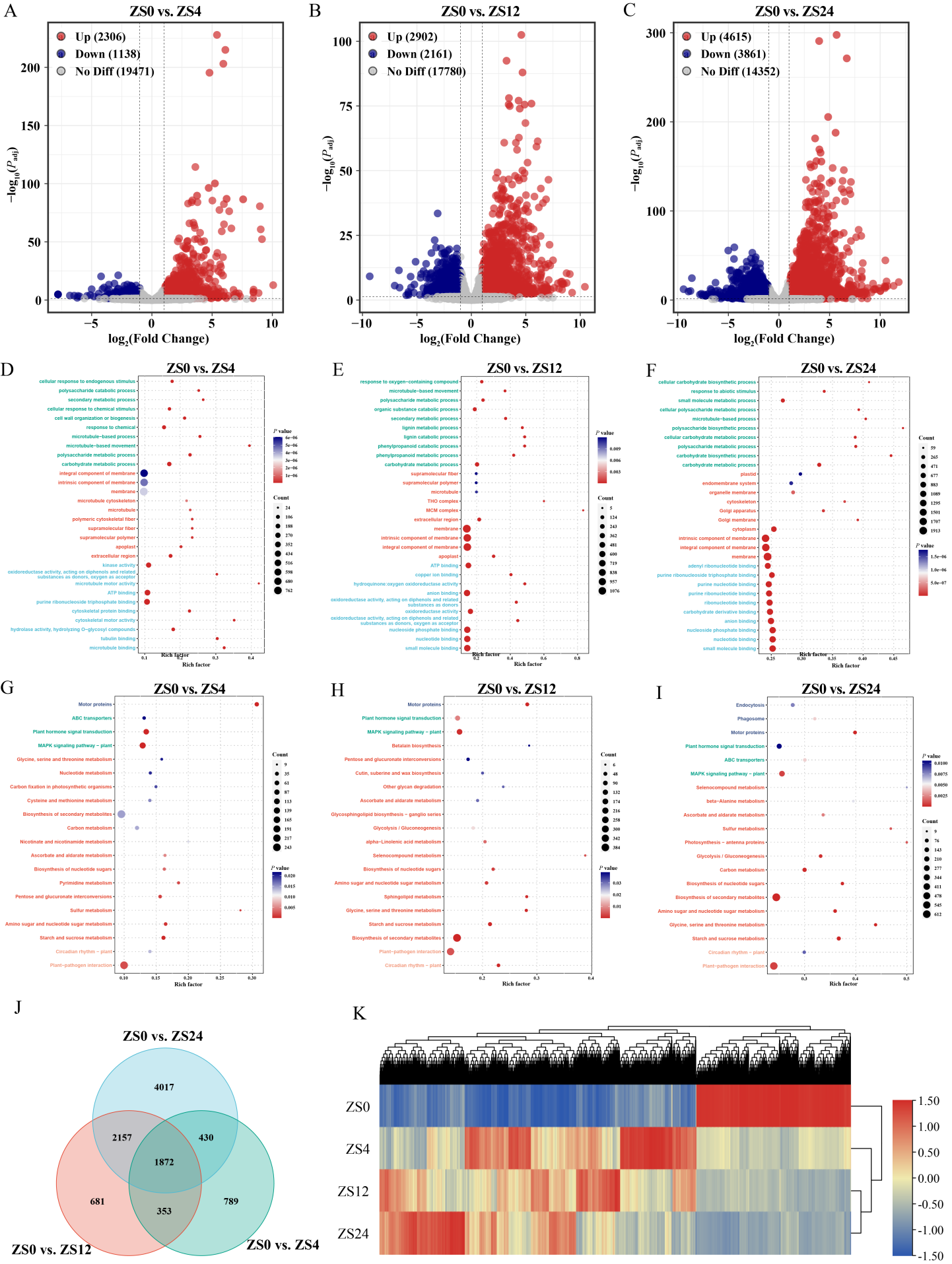


**Supplementary Figure 4.** Transcriptome dynamics and functional enrichment in *R*. *rugosa* one-year-old stem under cold stress (4°C).

(A–C) Number of DEGs identified in one-year-old stem of *R*. *rugosa* under cold stress (4°C) at 4 h, 12 h, and 24 h compared to the 0 h control. (D–F) Bubble charts depicting GO functional enrichment results for the DEGs in stem at 4 h, 12 h, and 24 h of cold stress. The figure shows the top ten enriched terms from each GO category: biological processes (green), cellular components (red), and molecular functions (blue). Full term lists are available in Supplementary Table 11–13. (G–I) Bubble charts presenting enriched KEGG pathways for DEGs from shoot tissue at discrete cold-stress time points (4 h, 12 h, 24 h). Comprehensive pathway annotations are provided in Supplementary Table 14–16. (J) Venn diagram identifying a shared set of 1,872 DEGs regulated across all cold-stress time points (4 h, 12 h, 24 h) in the shoots. (K) Hierarchical clustering heatmap displaying the expression patterns of the core 1,872 DEGs.


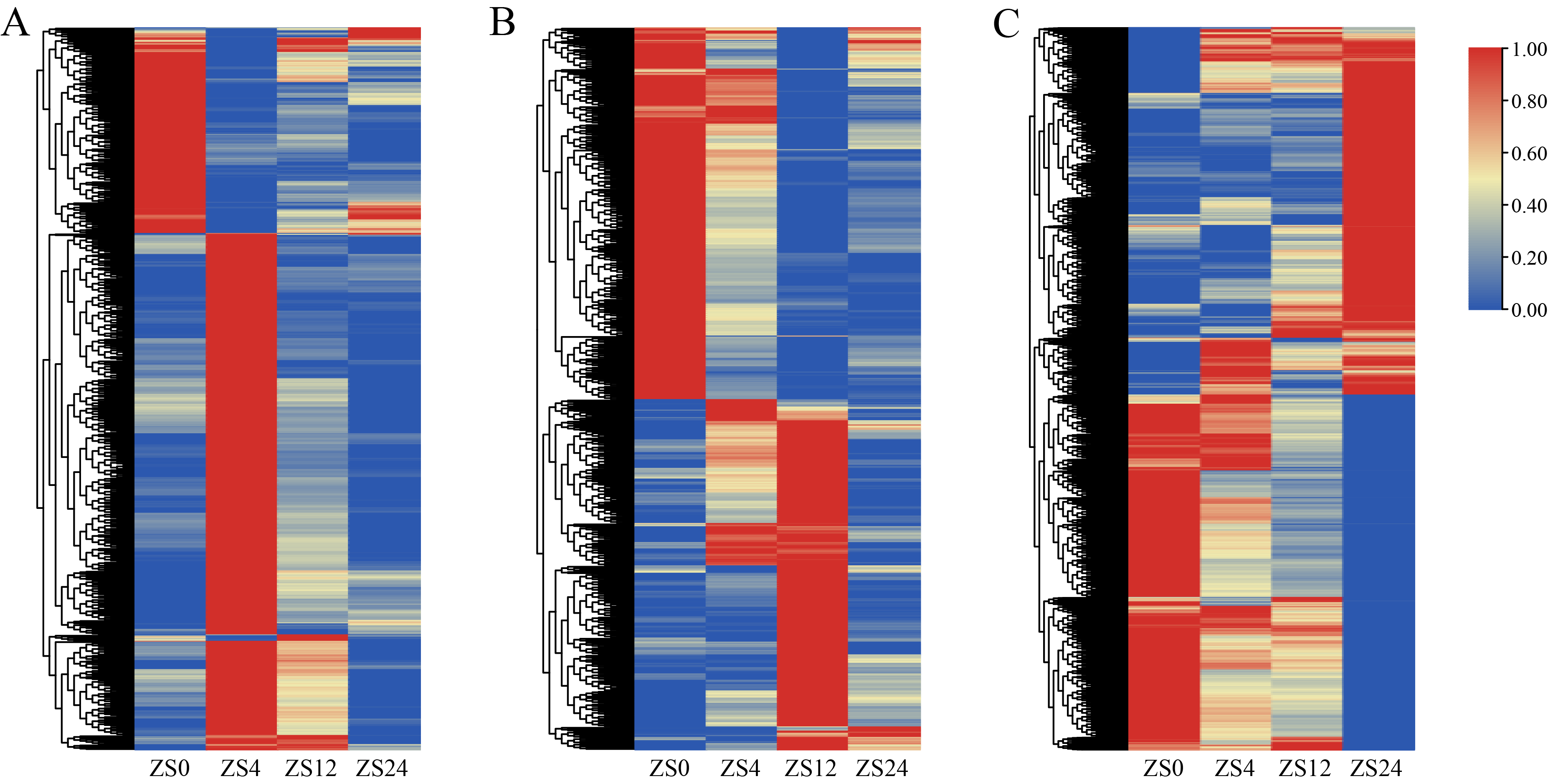


**Supplementary Figure 5.** Hierarchical clustering of time point-specific DEGs in *R*. *rugosa* one-year-old stem under cold stress (4°C).

(A) Hierarchical clustering heatmap of 789 DEGs specifically identified in stem after 4 h of cold stress (4°C). (B) Hierarchical clustering heatmap of 681 DEGs unique to the 12 h cold stress. (C) Hierarchical clustering heatmap of 4,017 DEGs specifically induced by 24 h of cold stress.


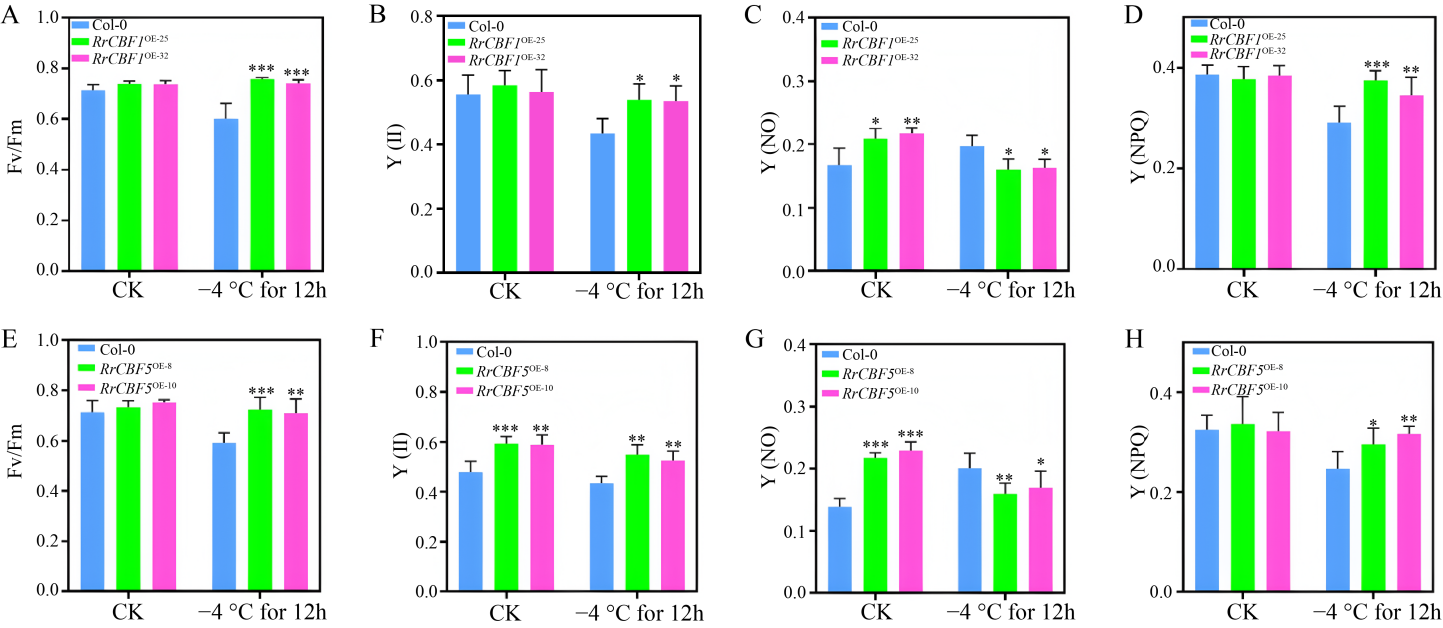


**Supplementary Figure 6. Chlorophyll fluorescence in WT and *RrCBF1*/*RrCBF5*-OE**Arabidopsis**under control (22°C) and cold stress (−4°C).**

**(A–H)** Chlorophyll fluorescence parameters—including the Fv/Fm (A, E), Y(II) (B, F), Y(NO) (C, G), and Y(NPQ) (D, H)—were measured in wild-type (Col-0) and transgenic Arabidopsis lines overexpressing RrCBF1 (A–D) or RrCBF5 (E–H) under control (22°C) and cold stress (−4°C for 12 h) conditions. Values are means ± SD (*n* = 12 biologically independent plants). Asterisks denote statistically significant differences compared to the wild-type at the corresponding temperature (*P < 0.05; **P < 0.01; ***P < 0.001; two-tailed Student’s *t*-test).
